# Heat Adaptation for Females: A Systematic Review and Meta-Analysis of Physiological Adaptations and Exercise Performance in the Heat

**DOI:** 10.1007/s40279-023-01831-2

**Published:** 2023-05-24

**Authors:** Monica K. Kelly, Steven J. Bowe, William T. Jardine, Dominique Condo, Joshua H. Guy, Rodney J. Snow, Amelia J. Carr

**Affiliations:** 1 Centre for Sport Research, Deakin University, 221 Burwood Highway, Burwood, VIC 3125 Australia; 2 Deakin Biostatistics Unit, Faculty of Health, Deakin University, Burwood, VIC Australia; 3 Faculty and School of Health, Victoria University of Wellington, Kelburn, Wellington, New Zealand; 4 School of Health, Medical and Applied Sciences, Central Queensland University, Cairns, QLD Australia; 5 Institute for Physical Activity and Nutrition, Deakin University, Burwood, VIC Australia

## Abstract

**Background:**

Heat adaptation regimes are used to prepare athletes for exercise in hot conditions to limit a decrement in exercise performance. However, the heat adaptation literature mostly focuses on males, and consequently, current heat adaptation guidelines may not be optimal for females when accounting for the biological and phenotypical differences between sexes.

**Objectives:**

We aimed to examine: (1) the effects of heat adaptation on physiological adaptations in females; (2) the impact of heat adaptation on performance test outcomes in the heat; and (3) the impact of various moderators, including duration (minutes and/or days), total heat dose (°C^.^min), exercise intensity (kcal^.^min^−1^), total energy expended (kcal), frequency of heat exposures and training status on the physiological adaptations in the heat.

**Methods:**

SPORTDiscus, MEDLINE Complete and Embase databases were searched to December 2022. Random-effects meta-analyses for resting and exercise core temperature, skin temperature, heart rate, sweat rate, plasma volume and performance tests in the heat were completed using Stata Statistical Software: Release 17. Sub-group meta-analyses were performed to explore the effect of duration, total heat dose, exercise intensity, total energy expended, frequency of heat exposure and training status on resting and exercise core temperature, skin temperature, heart rate and sweat rate. An explorative meta-regression was conducted to determine the effects of physiological adaptations on performance test outcomes in the heat following heat adaptation.

**Results:**

Thirty studies were included in the systematic review; 22 studies were meta-analysed. After heat adaptation, a reduction in resting core temperature (effect size [ES] =  − 0.45; 95% confidence interval [CI] − 0.69, − 0.22; *p* < 0.001), exercise core temperature (ES =  − 0.81; 95% CI − 1.01, − 0.60; *p* < 0.001), skin temperature (ES =  − 0.64; 95% CI − 0.79, − 0.48; *p* < 0.001), heart rate (ES =  − 0.60; 95% CI − 0.74, − 0.45; *p* < 0.001) and an increase in sweat rate (ES = 0.53; 95% CI 0.21, 0.85; *p* = 0.001) were identified in females. There was no change in plasma volume (ES = − 0.03; 95% CI − 0.31, 0.25; *p* = 0.835), whilst performance test outcomes were improved following heat adaptation (ES = 1.00; 95% CI 0.56, 1.45; *p* < 0.001). Across all moderators, physiological adaptations were more consistently observed following durations of 451–900 min and/or 8–14 days, exercise intensity ≥ 3.5 kcal^.^min^−1^, total energy expended ≥ 3038 kcal, consecutive (daily) frequency and total heat dose ≥ 23,000 °C^.^min. The magnitude of change in performance test outcomes in the heat was associated with a reduction in heart rate following heat adaptation (standardised mean difference =  − 10 beats^.^min^−1^; 95% CI − 19, − 1; *p* = 0.031).

**Conclusions:**

Heat adaptation regimes induce physiological adaptations beneficial to thermoregulation and performance test outcomes in the heat in females. Sport coaches and applied sport practitioners can utilise the framework developed in this review to design and implement heat adaptation strategies for females.

**Supplementary Information:**

The online version contains supplementary material available at 10.1007/s40279-023-01831-2.

## Key Points

**Table:** 

Females achieve physiological adaptations to heat adaptation regimes, including a reduction in resting and exercise core temperature, skin temperature, heart rate and an increase in sweat rate, but no change in plasma volume.
Performance test outcomes, including mean power and time to exhaustion, are improved in females following heat adaptation regimes.
Physiological adaptations were more consistently observed in heat adaptation regimes across all moderators when utilising durations of 451–900 min and/or 8–14 days of heat exposure, exercise intensity ≥ 3.5 kcal^.^min^−1^, TEE ≥ 3038 kcal, completed via a consecutive (daily) frequency and total heat dose ≥ 23,000 °C^.^min.

## Introduction

The representation of females in sports science and sports medicine research has failed to parallel the increase in participation and popularity of women’s sport and exercise [1]. Recent publications highlight the substantial under-representation of females with just 4–13% of all sports science and sports medicine studies being female only [2–5]. In exercise thermoregulatory research published between 2010 and 2019, 5% of studies had female-only cohorts with mixed-sex cohorts (combined male and female populations) representing 25% of studies [6]. Whilst there has been an increase in the proportion of females in thermoregulation studies in the last decade [6], there remains a large under-representation of females in this research area.

There are clear anatomical, physiological and endocrinological differences between the sexes [7–10], which may contribute to differences in the thermoregulatory response of females to exercise in the heat [11, 12]. Such differences have also been reported to influence the time course [13], and set-point temperature of the thermoregulatory response [14, 15] of females compared to males. Heat acclimation (completed within a hot artificial environment) and heat acclimatisation (completed within a hot natural environment) are heat adaptation strategies that require repeated exposures to a heat stress to induce physiological adaptations that enable improved thermal balance when exercising in the heat [16, 17]. It is imperative for sport coaches and applied sport practitioners involved with preparing female athletes for competition in hot environments that they are aware of  the thermoregulatory adaptations and factors required to implement an optimal heat adaptation regime for these athletes.

Previous heat adaptation reviews and meta-analyses have provided a basis for the exploration of thermoregulatory differences between male and females during exercise in the heat [18–23]. However, to our knowledge, there has been no meta-analysis that has exclusively investigated the physiological responses of females to heat adaptation protocols and subsequent impact on exercise performance. Therefore, the primary aim of this review was to systematically examine the effects of heat adaptation on the physiological variables related to thermoregulation including resting and exercise core temperature (*T*_core_), skin temperature (*T*_sk_), heart rate (HR), sweat rate (SR) and plasma volume (PV) in females. The secondary aim was to explore the impact of heat adaptation strategies on performance test outcomes in the heat in females. The tertiary aim was to examine the impact of various moderators including duration of heat exposure, total heat dose, exercise intensity (EI), total energy expended (TEE), frequency of heat exposures and training status on the physiological adaptations and performance test outcomes of females to the heat.

## Methods

This systematic review was designed in accordance with the Preferred Reporting Items for Systematic Reviews and Meta-Analyses (PRISMA) statement guidelines (Appendix S1 of the Electronic Supplementary Material [ESM]) [24].

### Inclusion and Exclusion Criteria

Consideration of the Population, Intervention, Comparison, Outcomes and Study Design (PICOS) was used to determine the parameters of the review. The inclusion criteria for this review involved healthy adult females of reproductive age, 18–50 years (*population)* who undertook a heat adaptation regime, including exercise or non-exercise heat exposure *(intervention),* in which physiological and/or performance measures from pre- to post-regimes *(outcome*) were compared. Full-text studies, published in a peer-reviewed journal of English language, with the primary or secondary objective of assessing changes in physiological variables and/or exercise performance following heat adaptation regimes (*study designs)* were included. Studies were excluded if they met one of the following criteria: dehydration induced prior to any exercise and/or testing in the heat, participants aged < 18 and/or > 50 years, focusing on sedentary or clinical female populations, human male participants only, concurrent interventions that did not allow for identification or comparison of heat adaptations, and unpublished or review articles, theses and conference abstracts. This study was not pre-registered.

### Study Search and Identification

A systematic electronic literature search was undertaken using three online databases (SPORTDiscus, MEDLINE Complete, Embase). Searches were performed using free-text thesaurus terms in addition to keywords from existing relevant papers [18, 19]. Search terms were phrased and truncated as appropriate. A complete search strategy from one database is presented (SPORTDiscus: 5/08/2020) [Appendix S2 of the ESM]. Databases were searched from inception through to December 2022. Reference lists of all studies and relevant systematic reviews were manually examined based on inclusion and exclusion criteria to identify additional studies for this review.

### Data Selection, Extraction and Study Quality Assessment

#### Selection of Studies

Following the removal of duplicates, a two-phase screening process was undertaken by two independent reviewers (M.K.K. and W.T.J.) using freely available Covidence systematic review software (version 2636; Veritas Health Innovation, Melbourne, VIC, Australia). Phase one assessed the eligibility of the title and abstract against predetermined inclusion and exclusion criteria. Studies that had unclear suitability remained within the review, with a final decision reached at the next stage. In phase two, full-text papers were retrieved for all articles that progressed from phase one and assessed against exclusion criteria. A third reviewer (A.J.C.) resolved any conflicts in a consensus meeting that arose during both phase one and phase two of the screening process.

#### Data Extraction

Data extraction was conducted by one reviewer (M.K.K.) using a pre-determined template. Extracted information included (1) characteristics of the participants including age, sex, maximal oxygen consumption and training status; (2) type of study and heat adaptation regime employed including duration, frequency, modality of exercise, ambient temperature and humidity; (3) outcome measures(s) including performance test outcomes and physiological adaptations; and (4) major findings of the study. Exercise *T*_core_, *T*_sk_, HR, SR, PV, and performance test outcome values were extracted from two timepoints where available: (1) baseline and/or pre-exercise and (2) immediately post-exercise either during first/last heat exposure or during a heat stress test. In studies where any post-exercise heat exposure had a longer time to exhaustion (TTE) than baseline and/or pre-exercise heat exposure, physiological data were extracted at equivalent timepoints to baseline and/or pre-exercise to allow comparison. For performance test outcomes, specifically TTE, data were extracted at the timepoint when exercise was ceased to quantify any difference in TTE within heat adaptation regimes. Resting *T*_core_ was extracted from baseline and/or pre-exercise heat exposure before and following heat adaptation regimes.

Where only figures were presented in a study (rather than numerical data within text and/or table), data were extracted using Web-Plot Digitizer Software [25]. This method was undertaken for ten studies [13, 26–34]. When standard error was reported, these were converted to standard deviation (SD). In cases of missing SD, data were estimated from the average SD of other studies of similar design under guidelines presented in Cochrane (Sect. 6.5.2.7) [35]. For instances where papers had more than two experimental groups, the group completing heat adaptation exposure for a within-participant analysis was included and/or only one experimental heat group was included to avoid participants being duplicated in the analysis. In this instance, conditions of hot-dry (terminology used by individual studies) were chosen for inclusion. Where data were presented at multiple timepoints within an adaptation regime, for example, short-term heat acclimation (STHA) and medium-term heat acclimation (MTHA), the data points representing the entire heat adaptation regime, for example, MTHA were included. For instances of cross-over study designs, only the heat intervention [36] or the exercise intervention [37] were included to allow comparison to other data sets. Where data were incomplete, further information was requested from the authors. The studies were excluded if these authors did not respond after 4 weeks from initial inquiry. A summary of all extracted data from included studies is available in Appendix S3 of the ESM.

#### Heat Adaptation Regime Categorisations

To be able to explore the influence of various moderators on exercise performance and the physiological adaptation to heat adaptation regimes, the following definitions and categorisations were utilised.

1. Performance test outcomes following heat adaptation regimes. Based on a classification utilised in a previous review [19], a performance test outcome was defined to elicit a maximal intensity effort within a defined time, distance, repetition or endpoint.
2. Heat induction method utilised in performance test outcomes. We examined the effect of method of heat adaptation on performance test outcomes by grouping data into controlled hyperthermia, controlled work-rate, self-regulated and mixed-method approaches (exercise and non-exercise heat exposure) [18]. A detailed description of each heat adaptation induction method is provided in another review [21].
3. Exposure duration of heat adaptation. This review categorised both the number of days of training aligning to STHA, MTHA and long-term heat acclimation (LTHA) and/or total minutes of heat exposure. As such, studies have been classified using the following criteria: short-term heat acclimation STHA classified as a heat exposure of $\le$ 450 min and/or $\le$ 7 days (equivalent to ~ 65 min per day for 7 days); MTHA classified as exposure between 451 and 900 min and/or 8–14 days (~ 65 min per day for 14 days) and LTHA training classified as $\ge$ 901 min and/or $\ge$ 15 days’ heat exposure. This combined method utilises the existing categorisation based on days of exposure [38] with the addition of total exposure minutes per protocol.
4. Total heat dose was quantified as the product of total heat exposure time (minutes) and ambient temperature (°C) used during the heat adaptation regime (°C^.^min). Three heat doses were identified based on tertiles calculated from the data set of the included studies within this review, including: low (≤ 23,000 °C^.^min), intermediate (23,001–43,200 °C^.^min) and high (≥ 43,201 °C^.^min) heat doses. The concept for this variable has been adapted from research investigating altitude training [39] and a previous heat adaptation meta-analysis [40].
5. EI during heat adaptation was quantified from the relative oxygen consumption of each protocol (mL^.^kg^−1.^min^−1^) converted into calories (kcal^.^min^−1^). This was calculated using the established conversion of 1 L^.^min^−1^ of oxygen consumption to 5 kcal^.^min^−1^ [41]. Three EI categories were identified as low (1.5–3.4 kcal^.^min^−1^), intermediate (3.5–5.4 kcal^.^min^−1^) and high ($\ge$ 5.5 kcal^.^min^−1^), based on published EI classifications [41].
6. TEE within a heat adaptation protocol was calculated from the EI (kcal^.^min^−1^) multiplied by the total duration (minutes) of the heat adaptation regime. Three categories were identified from tertiles calculated from the data set and were classified as low (< 3037 kcal), intermediate (3038–6749 kcal) and high (≥ 6750 kcal) TEE.
7. Frequency of heat exposure was classified as consecutive (daily) and non-consecutive (non-daily). Consecutive exposure required daily heat sessions, whilst non-consecutive exposure had up to 3 days between each heat session.
8. Training status was classified according to a recently published participant classification framework [42]. Participants of included studies were retrospectively allocated to five possible tiers based on training volume/physical activity metrics, performance standards and skill level [42]. In summary, participants in Tier 1 were Recreationally Active, Tier 2 were Trained/Developmental, Tier 3 were Highly Trained/National, Tier 4 were Elite/International and Tier 5 were World Class. If a study failed to report sufficient information, the study was classified as ‘insufficient information’ rather than within a tier, whilst Tier 0 represented Sedentary populations that were not included within this review.
9. The percentage change in performance test outcomes following meta-analyses were retrospectively categorised as low, intermediate and high from tertiles calculated from the current data set. The categorisation was as follows: low ($\Delta \le$ 8%), moderate ($\Delta$ >9–33%) and high ($\Delta \ge$ 34%) change in exercise performance in the heat after heat adaptation.

#### Risk of Bias Assessment

Risk of bias was conducted for the studies included in the meta-analysis according to the Cochrane Collaboration’s recommendation for systematic reviews [43]. The categories for assessment included (1) sequence generation, (2) allocation concealment, (3) blinding of participants, (4) blinding of outcome data, (5) incomplete outcome data, (6) selective outcome reporting and (7) other sources of bias. A low risk of bias, high risk of bias or unclear risk of bias was assigned to each category. Two authors (M.K.K. and W.T.J.) independently assessed each category, with results cross-checked for agreeance.

#### Statistical Analyses

For studies that reported within-group changes between pre- and post-heat adaptation on resting and exercise *T*_core_, *T*_sk_, HR, SR, PV and performance, a random-effect meta-analysis was performed using Stata 17 (Stata Statistical Software: Release 17, 2021; StataCorp LLC, College Station, TX, USA) [44]. The process of calculating effect sizes from means and SDs was based on existing formulae [45]. A value of *r*, representing the correlation between pre- and post-scores, was set at 0.7, as this was considered as a reasonable approximation of the correlation [45]. Data were reported as mean, standardised mean difference, SD, Hedges’ *g* effect size (ES) and 95% confidence intervals (95% CIs). Effect size interpretation was used whereby: *g* ≤ 0.19 = trivial/negligible effect, *g* = 0.20–0.49 = small effect, *g* = 0.50–0.79 moderate effect and *g* ≥ 0.8 = large effect [46]. Statistical significance was set to an alpha level of *p* < 0.05. Heterogeneity, represented as a *I*^2^ statistic to quantify percentage of variation across the studies was calculated in Stata 17, whereby *I*^2^ < 25% was considered low heterogeneity, *I*^2^ = 25–50% was considered moderate heterogeneity and *I*^2^ > 50% was considered high heterogeneity [47]. Performance test outcomes were presented as percentage change (%change) from pre-post testing. A sub-group analysis was conducted to explore the effect of moderators of duration of the heat adaptation regime, total heat dose, EI, TEE, frequency of heat exposure, training status, and method of heat induction on performance outcomes and physiological variables. Sub-group analyses were conducted when two or more studies (*k* ≥ 2) met sub-group categorisation. If a between-group difference was identified (*p* < 0.05), a regression was completed to determine the location of the between-group difference. An explorative meta-regression was performed to determine the effects of physiological adaptations on performance test outcomes presented as percentage change (%change) following heat adaptation strategies. Risk of bias plots were developed utilising Robvis [48] and funnel plots for publication bias were produced in Stata 17 (StataCorp LLC) [Appendices S4 and S5 of the ESM]. Definitions and groupings of classifications, including performance tests and moderators are found in Sect. 2.3.3.

## Results

### Systematic Review Results

#### Search Results

**Fig. 1:**
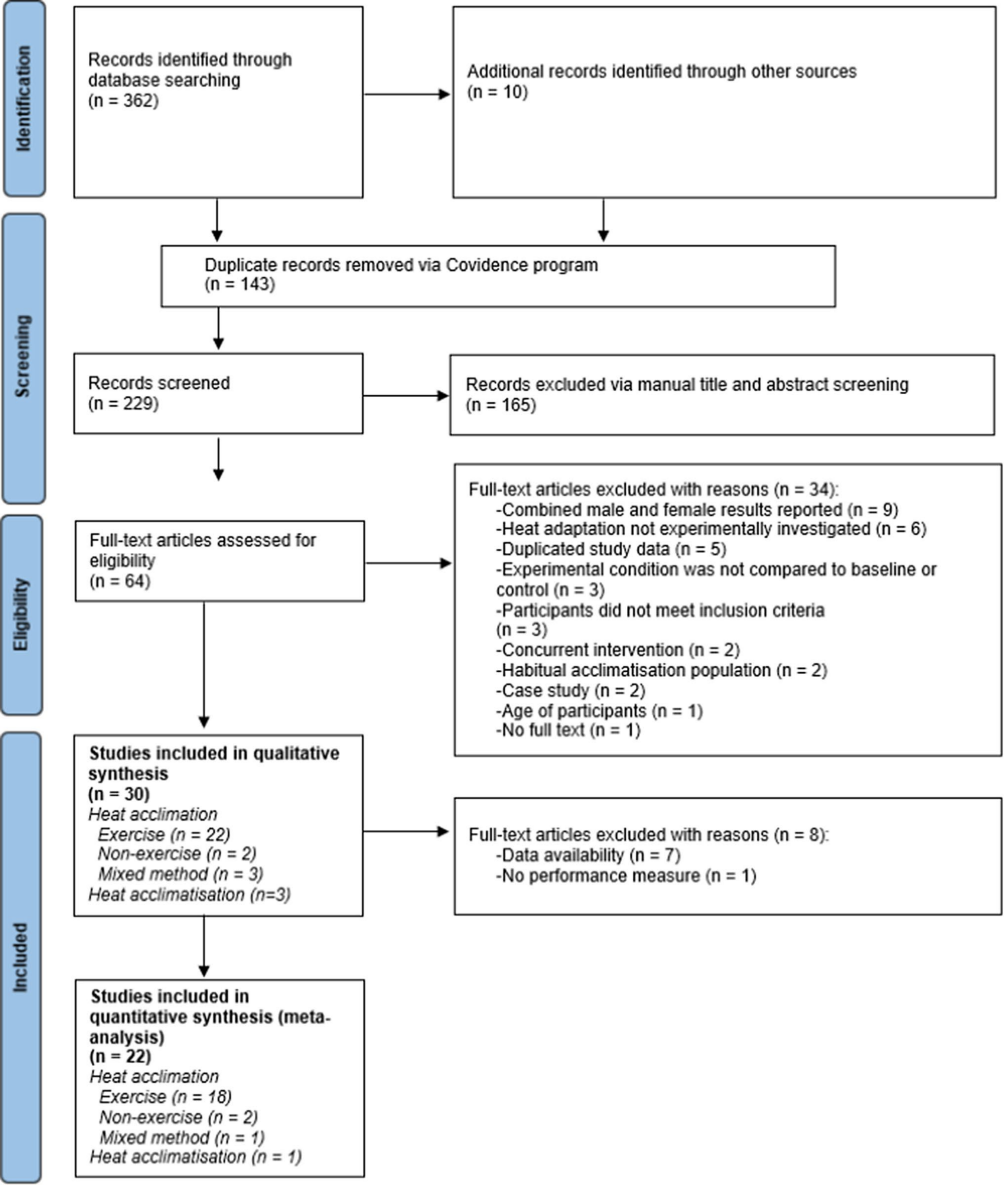
Preferred Reporting Items for Systematic Reviews and Meta-Analyses (PRISMA) flow diagram [24]

The search yielded 372 English-language articles. A further 143 studies were identified as duplicates and removed. A total of 229 articles were screened for title and abstract, with 165 excluded. Of the remaining 64 articles, 34 studies were excluded because of: combined male and female reported results (nine), heat adaptation not experimentally investigated (six), data duplicated in another publication (five), experimental condition not compared to a baseline or control (three), participants that did not meet inclusion criteria (three), concurrent intervention (two), participant population with habitual acclimatisation (two), case studies (two), participants’ age (one), and no full text (one). In total, 30 studies were eligible for inclusion in the systematic review and 22 studies were eligible for inclusion in the meta-analysis (Fig. 1). The main results and characteristics from the included studies are displayed in Table 1.

**Table 1:** Summary of the investigations utilising heat adaptation strategies in females included in this systematic review and meta-analysis

Study	Exercise modality and intensity	Number and duration of heat exposures	Days between	Heat exposure temperature (°C) and relative humidity (%)	Performance test type	Performance (% ∆)	*T*_core_ resting (°C ∆)	*T*_core_ exercise (°C ∆)	*T*_sk_ (°C ∆)	HR (b.min^−1^ ∆)	SR (% ∆)	PV (% ∆)
Alkemade et al. [49]	Cycling. Controlled hyperthermia (target *T*_core_ = 38.5 °C)	10 × 96.5 min	0	33 (65)	TTE	10	− 0.04	− 0.04	− 0.24	− 12	20	–
Avellini et al. [27]	Walking @ 5.6 km^.^h^−1^, 2% gradient (~ 30% $\dot{V}$˙O_2max_)	10 × 120 min	0	36	TTE	33	0.05	− 0.24	− 0.26	− 8	11	–
Barry et al. [50]^a^	Water immersion	7 × ~ 90 min	0	40–41	–	–	− 0.28	–	− 0.26	–	49	–
Buono et al. [51]	Walking @ 3 m^.^h^−1^; 3% gradient and cycling @ 60 W	8 × 120 min	0	35 (75)	–	–	–	− 0.40	− 0.30	− 8	2	–
Campbell et al. [37]	Cycling. Controlled hyperthermia (target *T*_core_ = 38.5 °C)	5 × 60 min	0	40 (52)	–	–	− 0.10	− 0.10	− 0.20	− 9	–	–
	Hot water immersion	5 × 60 min	0	40	–	–	–	–	–	–	–	–
	Sauna	5 × 60 min	0	55 (54)	–	–	–	–	–	–	–	–
Cleland et al. [52]	Walking @ ~ 5.6 km^.^h^−1^ 2.2–4% gradient	7 × ~ 60 min	0	42.2	TTE	54	–	–	–	–	–	–
Cohen and Gisolfi [34]	Walking @ ~ 5.6 km^.^h^−1^ (30–35% $\dot{V}$O_2max_)	8 × 240 min	0	45	TTE	23	− 0.35	− 0.61	− 0.29	− 14	–	–
Fein et al. [53]	Walking @ 5.2 km^.^h^−1^ 2.5% gradient	10 × 100 min	0^b^ 3^c^	46.5	TTE TTE	67 67	–	–	–	–	–	–
Frye and Kamon [26]	Walking @ 25–30% $\dot{V}$˙O_2max_	8–9 × 120 min	0	48	TTE	120	–	− 0.75	–	–	–	–
Garrett et al. [31]	Cycling. Controlled hyperthermia (target *T*_core_ = 38.5 °C)	5 × 90 min	0	39.5 (60)	MPO	4	–	− 0.20	− 0.50	–	–	–
Gore et al. [54]^d,e^	Rowing @ 18–22 strokes per min to 28–30 strokes per min	2 × 70 min up to 2 × 90 min per day over 4 weeks	0	32	–	–	–	–	–	–	–	–
Greenleaf et al. [33]	Cycling @ ~ 49% $\dot{V}$˙O_2peak_	12 × 120 min	0	40 (42)	–	–	–	–	–	− 21	24	6
Henderson et al. [55]^d^	Rugby 7’s specific training	5 × ~ 61 min	1	32 (58)	–	–	0.00	–	–	− 4	− 9	–
Horstman and Christensen [32]	Cycling @ 40% $\dot{V}$˙O_2max_	11 × 120 min	0	45	TTE	45	–	− 0.69	–	− 27	–	–
Kampmann et al. [56]^e^	Walking @ 4 km^.^h^−1^ (warm and humid)	15 × 122 min	0	37 (70)	–	–	–	–	–	–	–	–
	Walking @ 4 km^.^h^−1^ (hot and dry)	15 × 122 min	0	50 (15)	–	–	–	–	–	–	–	–
	Walking @ 4 km^.^h^−1^ (radiant heat)	15 × 122 min	0	25 (40)	–	–	–	–	–	–	–	–
Kirby et al. [57]	Cycling. Controlled hyperthermia (target *T*_core_ = 38.5 °C)	9 × 90 min	0	40 (30)	TT (MPO)	8	− 0.20	− 0.08	− 0.67	− 5	21	–
Kirby et al. [58]^a^	Sauna. Sedentary exposure	9–10 × 29 min	2–3	104 (5–9)	–	–	− 0.20	− 0.40	− 1.00	− 10	18	0
Mee et al. [13]	Cycling. Controlled hyperthermia (target *T*_core_ = 38.5 °C)	10 × 90 min	0	40 (40)	–	–	− 0.24	− 0.47	− 1.08	− 10	145	–
Mee et al. [36]^a^	Sauna: 20 min seated in vinyl suit. Cycling. Controlled hyperthermia (target *T*_core_ = 38.5 °C)	5 × 90 min	0	50 (30) 40 (40)	–	–	− 0.28	− 0.42	− 0.89	− 12	81	–
Meylan et al. [59]^d,e^	Field soccer training	5 × ~ 102 min	1	34.5 (53)	–	–	–	–	–	–	–	–
Moss et al. [60]	Cycling. Controlled hyperthermia (target *T*_core_ = 38.5 °C)	10 × 60 min	0	40 (50)	–	–	− 0.34	− 0.68	− 0.66	− 38	50	–
O’Toole et al. [28]	Cycling @ 50 rev^.^min^−1^, ~ 46% $\dot{V}$O_2max_	10 × 120 min	0	40 (50)	–	–	–	− 0.32	–	− 8	13	–
Pethick et al. [61]^e^	Intermittent exercise. Controlled hyperthermia (target *T*_core_ = 38.5 °C)	5 × 90 min	0	35 (36–46)	–	–	–	–	–	–	–	–
Philp et al. [62]^e,f^	Cycling or rowing ergometry	10 × 60 min	0	34 (55)	–	–	–	–	–	–	–	–
Sawka et al. [63] (HA)^e^	Walking @ ~ 5 km^.^h^−1^, 0% gradient	6 × 110 min	0	49 (20)	–	–	–	–	–	–	–	–
Sawka et al. [29] (Hypo)	Walking @ ~ 5 km^.^h^−1^, 0% gradient (~ 30% $\dot{V}$O_2max_) (hot and dry)	10 × 110 min	0	49 (20)	–	–	–	− 0.27	− 0.44	− 25	-4	–
Sawka et al. [29] (Hypo)^e^	Walking @ ~ 5 km^.^h^−1^, 0% gradient (~ 30% $\dot{V}$O_2max_) (hot and humid)	10 × 110 min	0	35 (79)	–	–	–	–	–	–	–	–
Shapiro et al. [15]	Walking @ ~ 5 km^.^h^−1^, 0% gradient	6 × 120 min	0	49 (20)	–	–	–	− 0.70	− 0.55	− 27	− 3	–
Stephenson et al. [64]^a^	Cycling. Controlled HR (~ 80% HR_max_) and sedentary heat exposure	8 × 45–90 min	0	35 (63)	TT (MPO)	6	0.03	− 0.71	− 0.23	− 10	28	–
Sunderland et al. [30]	Intermittent sprint exercise (50– 85% $\dot{V}$O_2max_)	4 × 38 min	1–2	30 (24)	TTE	33	0.00	− 0.30	–	− 9	–	− 2
Wyndham et al. [65]^e^	Bench stepping exercise @ ~ 1 L.min^−1^	20 × 240 min	0	34	–	–	–	–	–	–	–	–

*HA* heat acclimation, *HR* heart rate, *HR*_*max*_ maximal heart rate, *Hypo* hypohydration, *L*^*.*^*min*^*−1*^ litres per minute, *m*^*.*^*h*^*−1*^ miles per hour, *min* minute(s), *MPO* mean power output, *PV* plasma volume, *Rev*^*.*^*min*^*−1*^ revolutions per minute, *SR* sweat rate, *T*_*core*_ core temperature, *T*_*sk*_ skin temperature, *TTE* time to exhaustion, *TT* time trial, $\dot{V}$˙O_*2max*_ maximal oxygen consumption, *V̇O*_*2peak*_ peak oxygen uptake.

^a^Includes sedentary heat exposure

^b^Consecutive

^c^Non-consecutive

^d^Acclimatisation

^e^Included in the systematic review only

^f^Combined male and female data

#### Characterisation of Participants

There were 235 female participants who undertook heat training, with an average age of 24 years (range of 18–46 years), and sample sizes between 3 and 20 participants (meta-analysed) (Appendix S6 of the ESM). The training statuses of participants undertaking heat adaptation regimes were recreationally active (Tier 1) [*k* = 2;* n* = 14], trained/developmental (Tier 2) [*k* = 4,* n* = 37], highly trained/national (Tier 3) [*k* = 2, *n* = 6], elite/international (Tier 4) [*k* = 3,* n* = 53] and ‘insufficient information’ [*k* = 19,* n* = 125]*.*

#### Characterisation of Studies

The included studies had a publication date range from 1965 to 2022. The temperature in which heat adaptation regimes occurred (mean ± SD) for all included studies was 42 °C ($\pm$ 13 °C) with a range of 25–104 °C (including sedentary sauna exposure) and relative humidity (%RH) of 44%RH ($\pm$ 19%RH) with a range of 5–79%RH (including sedentary sauna exposure) (Table 1). Of the included studies in the systematic review, the majority of studies artificially simulated hot conditions to induce heat acclimation (*k* = 27) compared with heat acclimatisation (*k* = 3). Of the 30 included studies, the following heat adaptation methods were utilised: controlled work rate (*k* = 16), controlled hyperthermia (*k* = 7), self-selected work rate (*k* = 3), controlled hyperthermia and non-exercise heat adaptation (*k* = 1), controlled HR and non-exercise heat adaptation (*k* = 1), non-exercise exposure in the heat and exercise in a temperate environment (*k* = 1) and non-exercise heat exposure only (*k* = 1). The average number of heat exposures per protocol was 9 ($\pm$ 4) sessions, with a range of 4–20 sessions. The average duration of heat sessions was 102 ($\pm$ 45 min), with a range of 29–240 min. Of the 30 included studies, the majority utilised a consecutive (daily) approach to heat adaptation (*k* = 25), compared to a non-consecutive approach (non-daily) (*k* = 4) and combined consecutive and non-consecutive methods (*k* = 1). Of the 30 included studies, a variety of exercise modes were utilised including walking (*k* = 9), cycling (*k* = 9), cycling and non-exercise heat exposure (*k* = 2), combination of exercise methods (*k* = 3), team sport training (*k* = 2), rowing (*k* = 1), running (*k* = 1), running and non-exercise heat exposure (*k* = 1), and stepping (*k* = 1). Non-exercise heat exposure was also utilised (*k* = 1). Of the 30 included studies, performance test outcomes following heat adaptation regimes were measured in a variety of ways, including TTE (*n* = 11), power output (*n* = 3), 30–15 intermittent fitness test (*n* = 1), small-sided game performance [soccer] (*n* = 1), 4-min rowing time trial (*n* = 1) and studies where no performance test was completed (*n* = 13). Table 1 presents further information on the included studies’ characteristics.

#### Risk of Bias

**Fig. 2:**
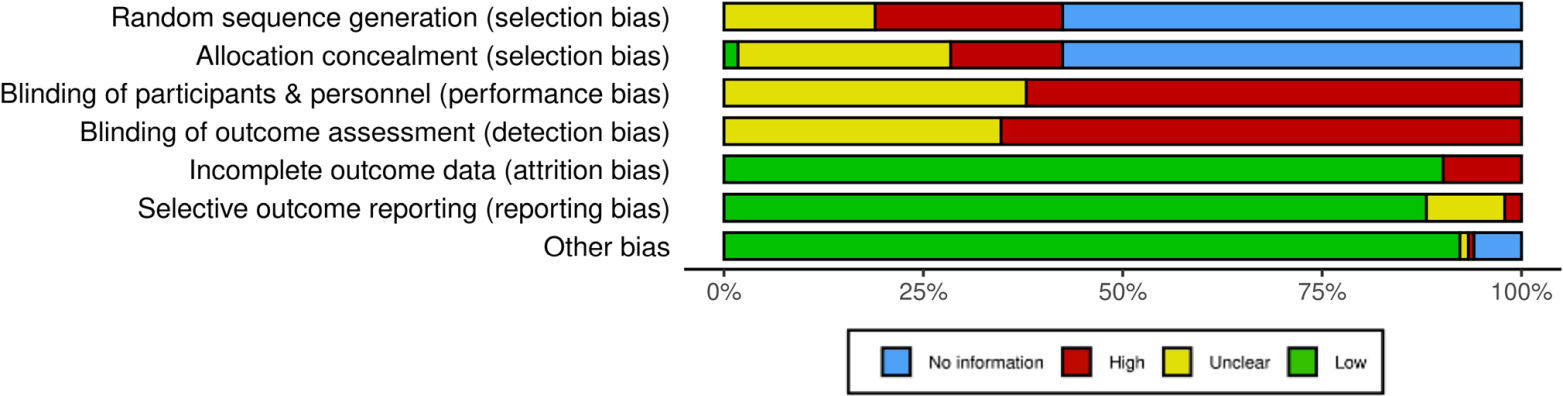
Risk of bias assessment

The included studies mostly had a low risk of bias, with outcome data clearly reported in the majority of studies (Fig. 2). Only the experimental heat intervention groups were included in the analysis, therefore random sequencing was not always applicable. Because of the nature of heat adaptation regimes, blinding of participants was not completed. Other bias was generated from the lack of menstrual cycle control within studies. For risk of bias of individual studies, including publication bias analyses, see Appendices S4 and S5 of the ESM.

### Meta-Analysis Results

#### Physiological Adaptations Following Heat Adaptation

**Fig. 3:**
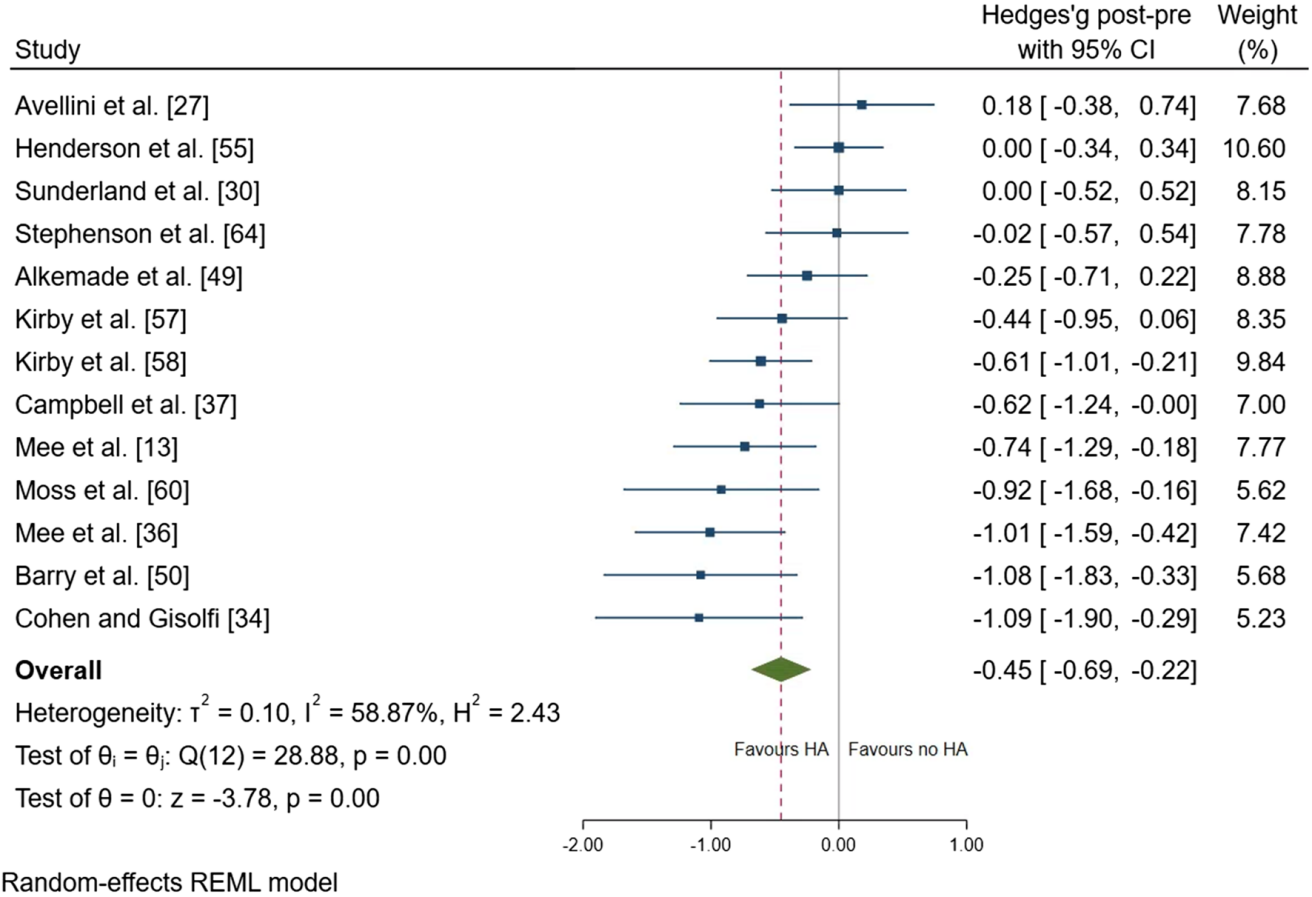
Resting core temperature plot. Data are presented as Hedges’ *g* and 95% confidence intervals (CIs). Effects to the left of 0 (solid line) indicate a reduction in resting core temperature, whereas effects to the right of 0 indicate no reduction in the resting core temperature with heat adaptation (HA) regimes. The red dotted line indicates the effect line of included studies within the Forest plot

**Fig. 4:**
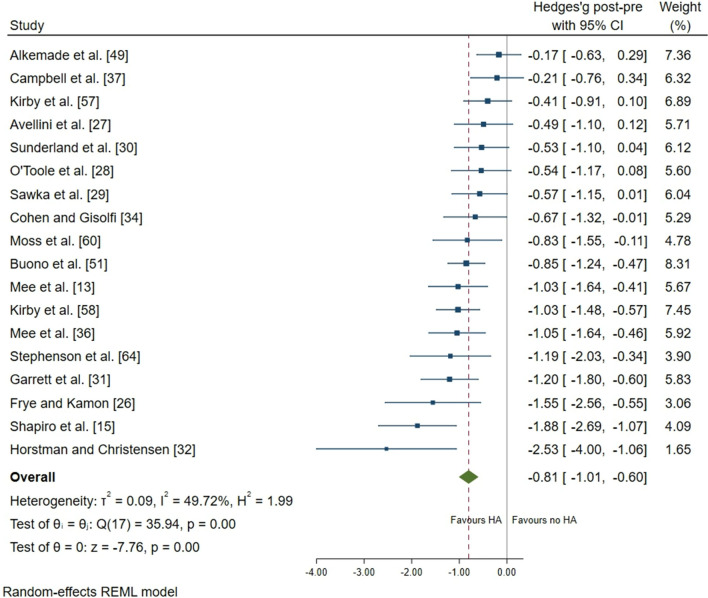
Exercise core temperature Forest plot. Data are presented as Hedges’ *g* and 95% confidence intervals (CIs). Effects to the left of 0 (solid line) indicate a reduction in exercise core temperature, whereas effects to the right of 0 indicate no reduction in exercise core temperature with heat adaptation (HA) regimes. The red dotted line indicates the effect line of included studies within the Forest plot

**Fig. 5:**
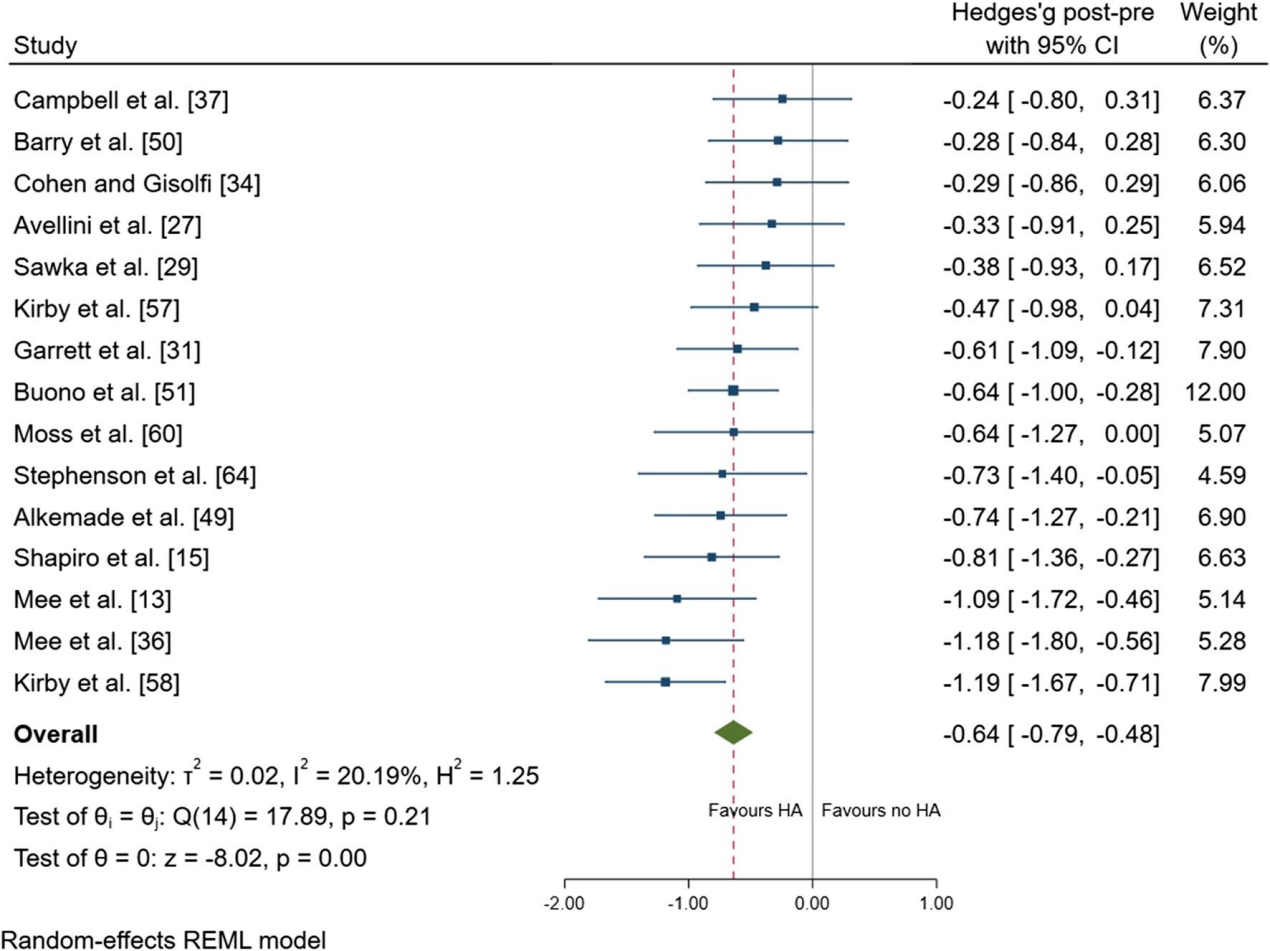
Skin temperature forest plot. Data are presented as Hedges’ *g* and 95% confidence intervals (CIs). Effects to the left of 0 (solid line) indicate a reduction in skin temperature, whereas effects to the right of 0 indicate no reduction in skin temperature with heat adaptation (HA) regimes. The red dotted line indicates the effect line of included studies within the Forest plot

**Fig. 6:**
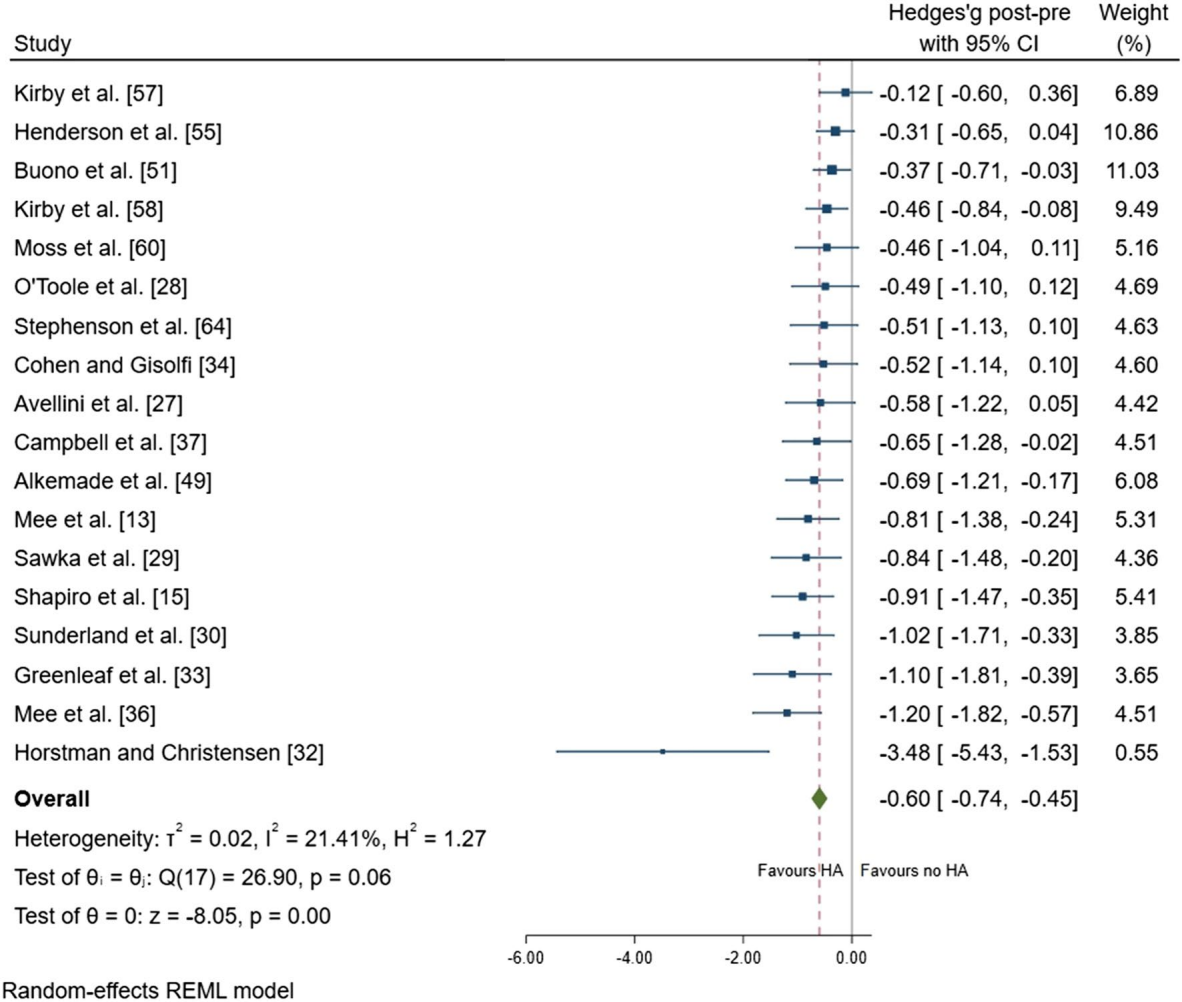
Heart rate forest plot. Data are presented as Hedges’ *g* and 95% confidence intervals (CIs). Effects to the left of 0 (solid line) indicate a reduction in heart rate, whereas effects to the right of 0 indicate no reduction in heart rate with heat adaptation (HA) regimes. The red dotted line indicates the effect line of included studies within the Forest plot

**Fig. 7:**
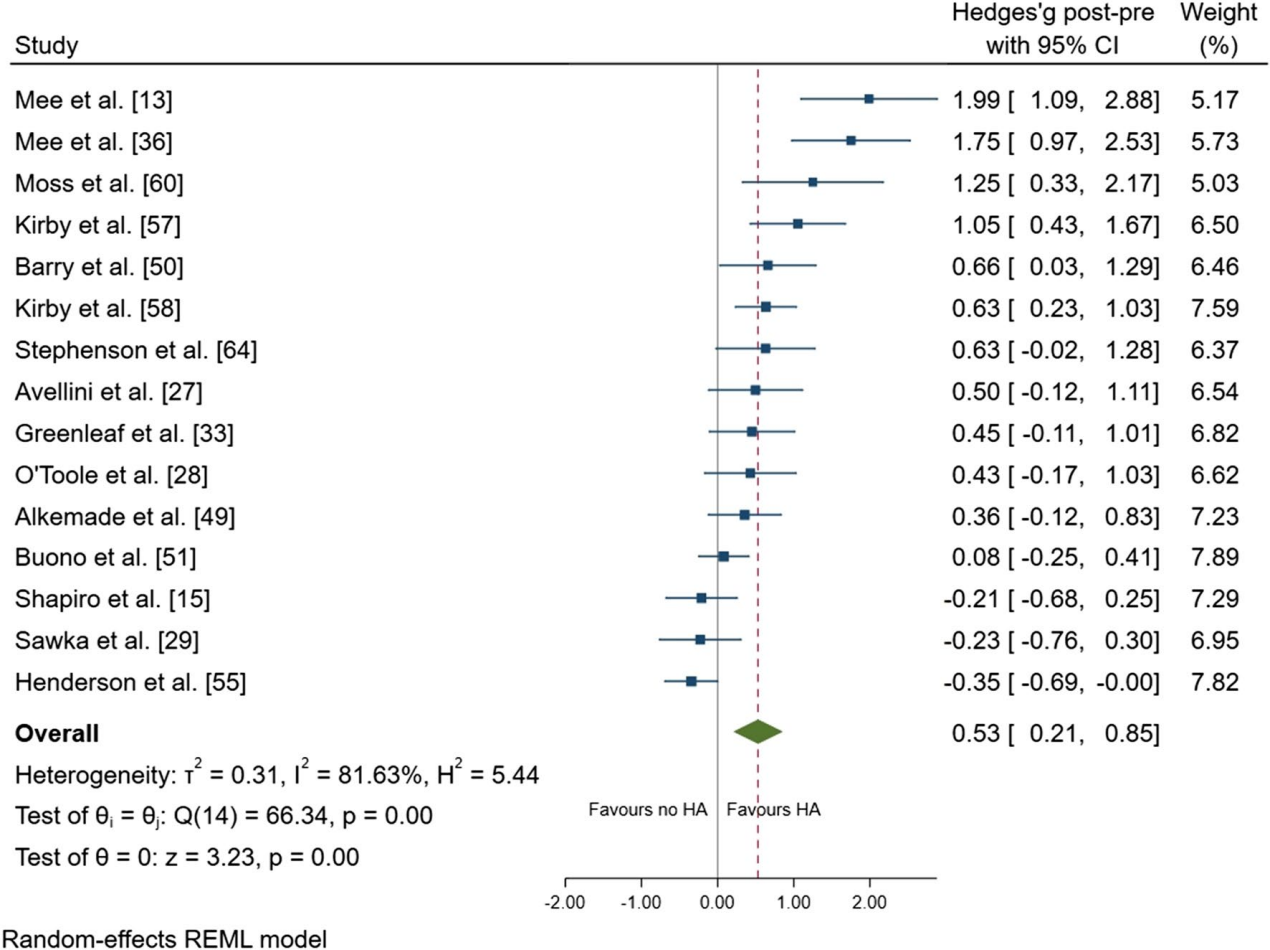
Sweat rate forest plot. Data are presented as Hedges’ *g* and 95% confidence intervals (CIs). Effects to the left of 0 (solid line) indicate a reduction in sweat rate, whereas effects to the right of 0 indicate an increase in sweat rate with heat adaptation (HA) regimes. The red dotted line indicates the effect line of included studies within the Forest plot

Following heat adaptation regimes, there was a small effect for a reduction in resting *T*_core_ (mean ± SD =  − 0.15 ± 0.15 °C; ES =  − 0.45; 95% CI − 0.69, − 0.22; *p* < 0.001) (Fig. 3). There was a large effect of a reduction in exercise *T*_core_ after heat adaptation regimes (mean =  − 0.41 ± 0.24 °C; ES =  − 0.81; 95% CI − 1.01, − 0.60; *p* < 0.001) (Fig. 4). There was a moderate effect for a reduction in *T*_sk_ (mean =  − 0.50 ± 0.30 °C; ES =  − 0.64; 95% CI − 0.79, − 0.48; *p* < 0.001) (Fig. 5), and HR (mean =  − 14 ± 9 beats.min^−1^; ES =  − 0.60; 95% CI − 0.74, − 0.45; *p* < 0.001) (Fig. 6). There was a moderate effect for an increase in SR (mean =  + 30 ± 40%; ES = 0.53; 95% CI 0.21, 0.85; *p* = 0.001) (Fig. 7) after a period of heat adaptation. There was a trivial non-significant change in PV after heat adaptation (mean =  − 1 ± 4%; ES = − 0.03; 95% CI − 0.31, 0.25; *p* = 0.835) [Appendix S7 of the ESM].

#### Performance Test Outcomes in the Heat Following Heat Adaptation

**Fig. 8:**
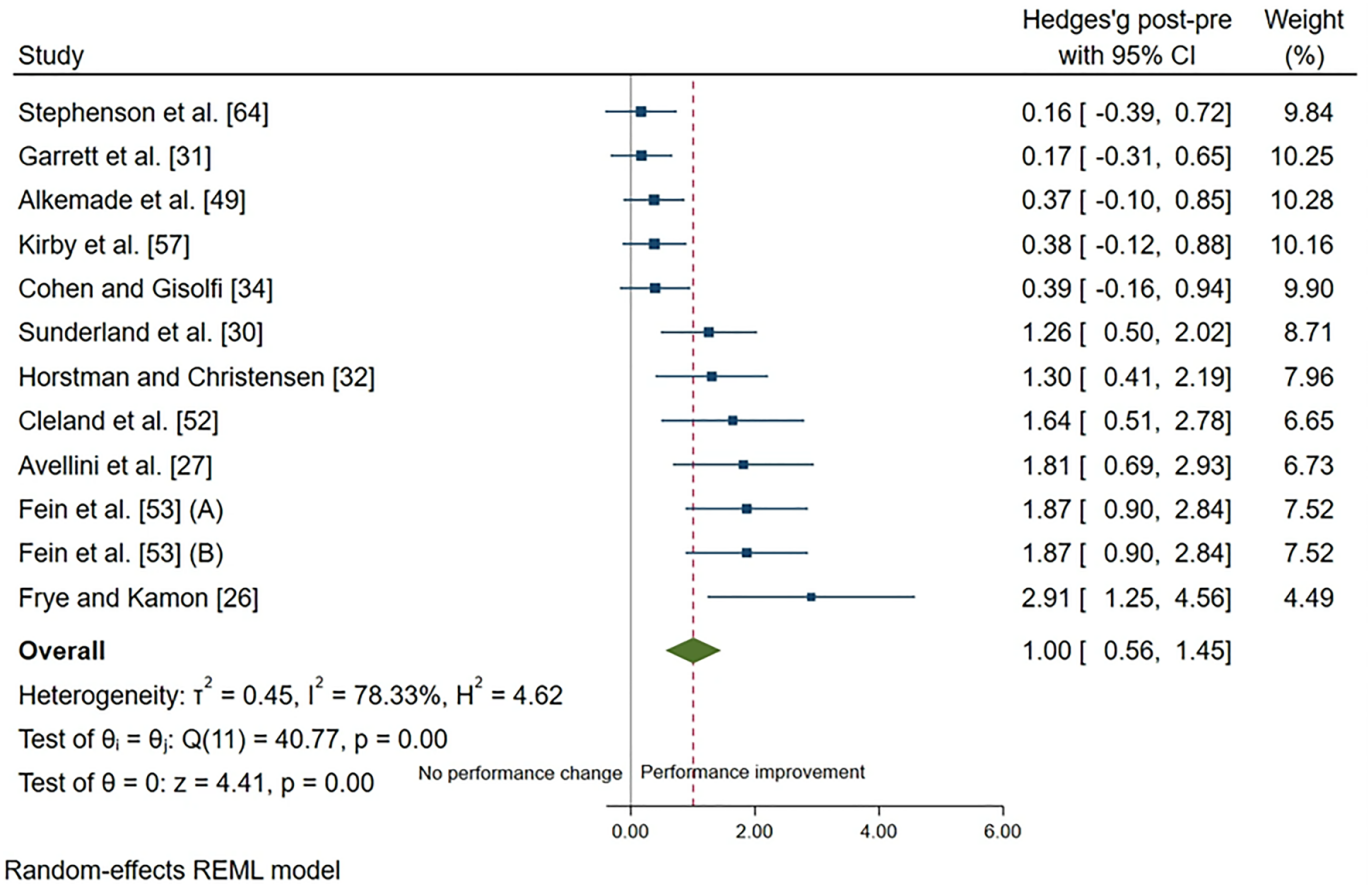
Performance test outcomes in the heat forest plot. Data are presented as Hedges’ *g* and 95% confidence intervals (CIs). Effects to the left of 0 (solid line) indicate no change in performance test outcomes, whereas effects to the right of 0 indicate an improvement in performance test outcomes with heat adaptation regimes. The red dotted line indicates the effect line of included studies within the Forest plot. Results presented for Fein et al. (**A**) refer to participants exposed to heat adaptation regimes on a consecutive (daily) basis, whilst results for Fein et al. (**B**) refer to participants exposed to heat adaptation regimes on a non-consecutive (non-daily) basis

After heat adaptation, there was a large effect for an improvement in performance test outcomes in the heat (mean = 39 ± 34%; ES = 1.00; 95% CI 0.56, 1.45; *p* < 0.001) (Fig. 8).

### Factors Influencing Performance Test Outcomes in the Heat Following Heat Adaptation

A sub-group analysis identified no between-group difference when investigating moderators of duration of heat adaptation regime (*p* = 0.62), total heat dose (*p* = 0.67), EI (*p* = 0.28), TEE (*p* = 0.86), and frequency of heat exposure (*p* = 0.14) on performance test outcomes following heat adaptation regimes. There was a between-group difference for performance test outcomes and method of heat induction (*p* = 0.01), with a greater effect on performance test outcomes with a controlled work rate compared to controlled hyperthermia (Table 2).

**Table 2:** Sub-group analyses summary of performance test outcomes in the heat, including mean power and time to exhaustion

Moderator	*k*	*n*	Mean (± SD) %	Effect size (95% CI)	*p-*value
Duration					
STHA	3	17	30 (25)	0.93 (0.03, 1.82)	0.043
MTHA	5	33	32 (32)	0.84 (0.13, 1.54)	0.020
LTHA	4	18	55 (44)	1.42 (0.44, 2.39)	0.004
Total heat dose					
Low	4	21	24 (24)	0.70 (0.00, 1.40)	0.050
Int	3	21	17 (14)	0.70 (− 0.05, 1.46)	0.067
High	3	14	63 (51)	1.35 (0.03, 2.67)	0.045
EI					
Low	–	–	–	–	–
Int	3	14	59 (53)	1.54 (0.11, 2.97)	0.034
High	6	34	26 (21)	0.72 (0.27, 1.17)	0.002
TEE					
Low	3	13	31 (24)	0.93 (0.04, 1.83)	0.041
Int	4	25	43 (53)	1.17 (0.08, 2.26)	0.035
High	2	10	34 (16)	0.78 (− 0.11, 1.66)	0.084
Frequency					
Con	10	56	37 (36)	0.91 (0.41, 1.40)	< 0.001
Non-con	2	12	50 (24)	1.49 (0.89, 2.09)	< 0.001
Training status					
Tier 1	1	9	–	–	–
Tier 2	2	12	7 (1)	0.28 (− 0.09, 0.66)	0.134
Heat induction method					
CH	3	25	7 (3)	0.31 (0.03, 0.59) ^a^	0.031
CWR	7	33	58 (32)	1.53 (0.94, 2.11)	< 0.001
Self-paced	1	6	–	–	–
Mixed	1	4	–	–	–

Positive effect sizes indicate findings in favour of an improvement in performance test outcomes of mean power and time to exhaustion with heat adaptation regimes Negative effect sizes indicate findings not in favour of an improvement in performance test outcomes of mean power and time to exhaustion with heat adaptation regimes

*CH* controlled hyperthermia, *CI* confidence interval, *Con* consecutive exposure, *CWR* controlled work rate, *EI* exercise intensity, *Int* intermediate exposure, *k* studies, *LTHA* long-term heat acclimation, *MTHA* medium-term heat acclimation, *n* participants, *Non-con* non-consecutive exposure, *SD* standard deviation, *STHA* short-term heat acclimation, *TEE* total energy expended

^a^Between-group difference (*p* = 0.01) for CH and CWR

The within-group analyses demonstrated that there was a large effect for an improvement in performance test outcomes after STHA, MTHA and LTHA (Table 2). There was a moderate non-significant effect for an improvement in performance test outcomes after a low and intermediate total heat dose (Table 2). There was a large effect for a high total heat dose exposure and an improvement in performance test outcomes (Table 2). After heat adaptation, a large effect for an improvement in performance test outcomes was identified with intermediate EI, and a moderate effect for high EI (Table 2). There were insufficient studies (*k* = 0) to investigate a low EI and performance test outcomes (Table 2). There was a large effect for an improvement in performance test outcomes with low and intermediate TEE, and a moderate, but non-significant effect following a high TEE heat adaptation regime (Table 2). There was a large effect for an improvement in performance test outcomes after a consecutive (daily) and non-consecutive (non-daily) heat exposure (Table 2). There was a small, but non-significant effect for female participants of Tier 2 (Trained/Developmental) classification and performance test outcomes (Table 2). There were insufficient studies (*k* = 1) to investigate female participants of Tier 1 (Recreationally Active) classification and performance test outcomes (Table 2). There were studies with participants of Tier 3 and Tier 4 calibre in this systematic review; however, these studies did not progress to the meta-analysis and are therefore not included in this investigation (k < 2). There was a small effect for an improvement in performance test outcomes and a controlled hyperthermia regime, with a large effect identified for a controlled work-rate approach (Table 2). There were insufficient studies to analyse a self-regulated and a mixed-method approach (*k* = 1), respectively.

### Factors Influencing Heat Adaptation

A sub-group analysis by study design represented as comparing (1) baseline and/or pre-exercise data with (2) immediately post-exercise data obtained either during the first/last heat exposure or during a heat stress test identified no between-group differences between first/last exposure and heat stress test for resting *T*_core_ (*p* = 0.51), exercise *T*_core_ (*p* = 0.99), *T*_sk_ (*p* = 0.22), HR (*p* = 0.85) and SR (*p* = 0.09). Consequently, the physiological data from both these types of exercise tests were pooled for all subsequent sub-group analyses and presented in Tables 3 and 4.

**Table 3:** Resting and exercise core temperature and skin temperature sub-group analyses summary

Moderator	Resting core temperature					Exercise core temperature						Skin temperature						
	*k*	*n*	Mean (± SD)	Effect size (95% CI)	*p-*value	*k*	*n*	Mean (± SD)	Effect size (95% CI)		*p-*value	*k*	*n*		Mean (± SD)	Effect size (95% CI)	*p-*value	
Duration																		
STHA	4	45	− 0.08 (0.10)	− 0.29 (− 0.64, 0.07)	0.111	4	37	− 0.25 (0.13)	− 0.75 (− 1.19, − 0.31)		0.001	3	31		− 0.55 (0.41)	− 0.69 (− 1.23, − 0.16)	0.011	
MTHA	6	37	− 0.22 (0.13)	− 0.66 (− 0.99, − 0.33)	< 0.001	6	41	− 0.51 (0.24)	− 1.00 (− 1.39, − 0.62)		< 0.001	7	46		− 0.62 (0.31)	− 0.72 (− 0.96, − 0.48)	< 0.001	
LTHA	3	17	− 0.11 (0.21)	− 0.33 (− 1.00, 0.34)	0.330	8	55	− 0.42 (0.25)	− 0.69 (− 0.97, − 0.40)		< 0.001	5	43		− 0.31 (0.08)	− 0.52 (− 0.74, − 0.30)	< 0.001	
Total heat dose																		
Low	5	42	− 0.07 (0.13)	− 0.30 (− 0.69, 0.10)	0.140	5	34	− 0.35 (0.24)	− 0.80 (− 1.20, − 0.40)		< 0.001	4	28		− 0.45 (0.32)	− 0.67 (− 1.04, − 0.30)	< 0.001	
Int	7	53	− 0.18 (0.14)	− 0.50 (− 0.79, − 0.22)	< 0.001	8	77	− 0.38 (0.25)	− 0.79 (− 1.10, − 0.47)		< 0.001	9	82		− 0.56 (0.32)	− 0.69 (− 0.89, − 0.49)	< 0.001	
High	1	4	–	–	–	5	22	− 0.53 (0.22)	− 0.93 (− 1.44, − 0.41)		< 0.001	2	10		− 0.37 (0.11)	− 0.33 (− 0.73, 0.06)	0.098	
EI																		
Low	0	0	–	–	–	2	15	− 0.49 (0.30)	− 1.19 (− 2.47, 0.08)		0.067	2	15		− 0.50 (0.08)	− 0.60 (− 1.02, − 0.17)	0.006	
Int	2	8	− 0.15 (0.28)	− 0.42 (− 1.67, 0.82)	0.506	3	12	− 0.53 (0.26)	− 0.77 (− 1.26, − 0.28)		0.002	2	8		− 0.28 (0.02)	− 0.31 (− 0.72, 0.10)	0.139	
High	8	52	− 0.15 (0.14)	− 0.46 (− 0.73, − 0.20)	0.001	11	80	− 0.38 (0.24)	− 0.69 (− 0.92, − 0.45)		< 0.001	8	66		− 0.53 (0.34)	− 0.68 (− 0.87, − 0.49)	< 0.001	
TEE																		
Low	3	15	− 0.02 (0.07)	− 0.18 (− 0.55, 0.19)	0.332	4	24	− 0.45 (0.30)	− 0.90 (− 1.62, − 0.18)		0.014	3	18		− 0.33 (0.19)	− 0.58 (− 0.96, − 0.21)	0.002	
Int	5	33	− 0.16 (0.16)	− 0.46 (− 0.87, − 0.04)	0.031	8	63	− 0.36 (0.26)	− 0.66 (− 0.91, − 0.41)		< 0.001	7	59		− 0.49 (0.25)	− 0.61 (− 0.81, − 0.42)	< 0.001	
High	2	12	− 0.30 (0.08)	− 0.85 (− 1.31, − 0.39)	< 0.001	4	20	− 0.52 (0.16)	− 0.89 (− 1.13, − 0.45)		<0.001	2	12		– 0.69 (0.56)	– 0.68 (− 1.47, 0.11)	0.092	
Frequency																		
Con	10	59	− 0.18 (0.15)	-0.55 (− 0.83, − 0.27)	< 0.001	16	111	− 0.42 (0.25)	− 0.81 (− 1.05, − 0.58)		< 0.001	14	104		− 0.47 (0.27)	− 0.59 (− 0.73, − 0.45)	< 0.001	
Non-con	3	40	− 0.07 (0.12)	-0.21 (− 0.62, 0.20)	0.312	2	22	− 0.35 (0.07)	− 0.81 (− 1.29, − 0.33)		0.001	1	16		–	–	–	
Training status																		
Tier 1	2	14	− 0.07 (0.04)	− 0.38 (− 0.75, − 0.01)	0.044	2	14	− 0.07 (0.04)	− 0.19 (− 0.54, 0.17)^a^		0.303	2	14		− 0.22 (0.03)	− 0.50 (− 0.99, − 0.01)	0.044	
Tier 2	4	37	− 0.16 (0.13)	− 0.52 (− 0.88, − 0.16)	0.005	4	37	− 0.40 (0.26)	− 0.87 (− 1.24, − 0.51)		< 0.001	4	37		− 0.70 (0.34)	− 0.89 (− 1.27, − 0.52)	< 0.001	

Negative effect sizes indicate findings in favour of a reduction in resting and exercise core temperature and skin temperature with heat adaptation regimes. Positive effect sizes indicate findings not in favour of a reduction in resting and exercise core temperature and skin temperature with heat adaptation regimes

*CI* confidence interval, *Con* consecutive exposure, *EI* exercise intensity, *Int* intermediate exposure, *k* studies, *LTHA* long-term heat acclimation, *MTHA* medium-term heat acclimation, *n* participants, *Non-con* non-consecutive exposure, *STHA* short-term heat acclimation, *TEE* total energy expended

^a^Between-group difference (*p *= 0.01) for Tier 1 and Tier 2 training status and exercise *T*_core_

**Table 4:** Heart rate and sweat rate sub-group analyses summary

Moderator	Heart rate					Sweat rate				
	*k*	*n*	Mean (± SD)	Effect size (95% CI)	*p-*value	*k*	*n*	Mean (± SD) %	Effect size (95% CI)	*p*-value
Duration										
STHA	4	46	− 8 (3)	− 0.48 (− 0.72, − 0.25)	< 0.001	2	35	5 (19)	0.14 (− 0.82, 1.10)	0.778
MTHA	6	41	− 17 (13)	− 0.65 (− 0.97, − 0.33)	< 0.001	7	46	53 (48)	0.96 (0.39, 1.53)	0.001
LTHA	8	57	− 15 (8)	− 0.62 (− 0.84, − 0.41)	< 0.001	6	49	11 (11)	0.22 (0.02, 0.42)	0.029
Total heat dose										
Low	5	43	− 9 (3)	− 0.68 (− 1.03, − 0.33)	< 0.001	3	32	33 (45)	0.64 (− 0.55, 1.83)	0.293
Int	8	77	− 15 (12)	− 0.50 (− 0.67, − 0.33)	< 0.001	9	82	35 (45)	0.62 (0.23, 1.01)	0.002
High	5	24	− 19 (8)	− 0.79 (− 1.11, − 0.47)	< 0.001	3	16	11 (14)	0.20 (− 0.25, 0.65)	0.374
EI										
Low	2	15	− 26 (2)	− 0.90 (− 1.32, − 0.47)	< 0.001	2	15	− 4 (1)	− 0.22 (− 0.57, 0.13) ^a^	0.218
Int	2	8	− 11 (4)	− 0.55 (− 0.99, − 0.11)	0.015	1	4	–	–	–
High	12	86	− 14 (10)	− 0.66 (− 0.87, − 0.45)	< 0.001	9	71	43 (45)	0.81 (0.40, 1.22)	< 0.001
TEE										
Low	4	24	− 14 (9)	− 0.78 (− 1.09, − 0.47)	< 0.001	2	13	13 (22)	0.18 (− 0.64, 1.00)	0.673
Int	7	59	− 15 (12)	− 0.56 (− 0.81, − 0.31)	< 0.001	7	59	26 (30)	0.62 (0.12, 1.12)	0.015
High	5	26	− 16 (8)	− 0.78 (− 1.08, − 0.47)	< 0.001	3	18	61 (73)	0.90 (− 0.05, 1.85)	0.064
Frequency										
Con	15	103	− 16 (10)	− 0.64 (− 0.81, − 0.47)	< 0.001	13	95	34 (41)	0.60 (0.26, 0.95)	0.001
Non-con	3	41	− 8 (3)	− 0.47 (− 0.76, − 0.19)	0.001	2	35	5 (19)	0.14 (− 0.82, 1.10)	0.778
Training status										
Tier 1	2	14	− 11 (2)	−0.68 (− 1.08, − 0.28)	0.001	1	9	–	–	–
Tier 2	4	37	− 9 (3)	− 0.54 (− 0.95, − 0.13)	0.010	4	37	37 (30)	0.95 (0.49, 1.42)	< 0.001

*CI* confidence interval, *Con* consecutive exposure, *EI* exercise intensity, *Int* intermediate, *k* studies, *LTHA* long-term heat acclimation, *MTHA* medium-term heat acclimation, *n* participants, *Non-con* non-consecutive exposure, *SD* standard deviation, *STHA* short-term heat acclimation, *TEE* total energy expended

Negative effect sizes indicate findings in favour of a reduction in heart rate with heat adaptation regimes. Positive effect sizes indicate findings not in favour of a reduction in heart rate with heat adaptation regimes. Positive effect sizes indicate findings in favour of an increase in sweat rate with heat adaptation regimes. Negative effect sizes indicate findings not in favour of an increase in sweat rate with heat adaptation regimes

^a^Between-group difference (*p* = 0.01) for low and high exercise intensity

#### Duration of Heat Exposure

There was no between-group difference across duration of heat exposure (STHA, MTHA and LTHA) for a reduction in resting *T*_core_ (*p* = 0.30), exercise *T*_core_ (*p* = 0.42), *T*_sk_ (*p* = 0.47) and HR (*p* = 0.61) and an increase in SR (*p* = 0.05). Within-group analyses demonstrated that after STHA heat adaptation regimes, there was a small effect for a reduction in HR, with a moderate effect for a reduction in exercise *T*_core_ and *T*_sk_ (Tables 3 and 4). There was a trivial non-significant increase in SR, and a small but non-significant reduction in resting *T*_core_ with STHA (Tables 3 and 4). Within MTHA heat adaptation regimes, there were moderate effects for a reduction in resting *T*_core_, *T*_sk_ and HR, and a large effect for a reduction in exercise *T*_core_ and an increase in SR (Tables 3 and 4). After LTHA regimes, there was a small effect for an increase in SR, and moderate effects for a reduction in *T*_sk_, exercise *T*_core_ and HR (Tables 3 and 4). There was a small non-significant effect for a reduction in resting *T*_core_ with LTHA regimes (Tables 3 and 4).

#### Total Heat Dose

There was no between-group difference in total heat dose (low, intermediate and high total heat dose) and a reduction in resting *T*_core_ (*p* = 0.21), and exercise *T*_core_ (*p* = 0.90), *T*_sk_ (*p* = 0.28), HR (*p* = 0.25) and SR (*p* = 0.37). Within-group analyses showed a moderate effect for a reduction in HR and *T*_sk_, and a large effect for a reduction in exercise *T*_core_ with a low total heat dose regime (Tables 3 and 4). There was a small non-significant effect for a reduction in resting *T*_core_ and a moderate non-significant effect for an increase in SR with low total heat dose regimes (Tables 3 and 4). After an intermediate total heat dose, there was a moderate effect for a reduction in resting *T*_core_, exercise *T*_core_, *T*_sk_ and HR and an increase in SR (Tables 3 and 4). After a high total heat dose adaptation regime, there was a moderate effect for a reduction in HR, and a large effect for a reduction in exercise *T*_core_ (Tables 3 and 4). There was a small non-significant effect for an increase in SR, and a small non-significant effect for a reduction in *T*_sk_ following a high total heat dose regime (Table 4). There were insufficient studies (*k* = 1) to investigate resting *T*_core_ and high total heat dose regimes.

#### Exercise Intensity

There was no between-group difference for EI (low, intermediate and high EI) and a reduction in resting *T*_core_ (*p* = 0.95), and exercise *T*_core_ (*p* = 0.72), *T*_sk_ (*p* = 0.27) and HR (*p* = 0.50). There was a between-group difference for EI and an increase in SR (*p* = 0.01), with no increase in SR with low EI and an increase in SR with high EI (Table 4). Within-group analyses found that after a low EI heat adaptation regime, there was a moderate effect for a reduction in *T*_sk_, and a large effect for a reduction in HR (Tables 3 and 4). Furthermore, there was a small non-significant effect for an increase in SR, and a large non-significant effect for a reduction in exercise *T*_core_ after a low EI regime (Tables 3 and 4). There were no studies (*k* = 0) to investigate resting *T*_core_ changes and low EI. After an intermediate EI heat adaptation regime, there was a moderate effect for a reduction in HR and a reduction in exercise *T*_core_ (Tables 3 and 4). There was a small non-significant effect for a reduction in resting *T*_core_ and *T*_sk_ whilst there were insufficient studies (*k* = 1) to explore an increase in SR following an intermediate EI regime (Tables 3 and 4). After a high EI adaptation regime, there was a small effect for a reduction in resting *T*_core_, a moderate effect for a reduction in exercise *T*_core_, *T*_sk_ and HR and a large effect for an increase in SR (Tables 3 and 4).

#### Total Energy Expended

There was no between-group difference for TEE (low, intermediate and high TEE) and a reduction in resting *T*_core_ (*p* = 0.08), and exercise *T*_core_ (*p* = 0.60), *T*_sk_ (p = 0.98), HR (*p* = 0.43) and SR (*p* = 0.50). Within-group analyses showed a moderate effect for a reduction in *T*_sk_ and a reduction in HR following a low TEE adaptation regime, and a large effect for a reduction in exercise *T*_core_ with low TEE (Tables 3 and 4). There was a trivial non-significant effect for a reduction in resting *T*_core_, and an increase in SR with low TEE (Tables 3 and 4). After an intermediate TEE adaptation regime, there was a small effect for a reduction in resting *T*_core_, and a moderate effect for a reduction in exercise *T*_core_, *T*_sk_ and HR, and an increase in SR (Tables 3 and 4). After a high TEE adaptation regime, there was a moderate effect for a reduction in HR, and a large effect for a reduction in resting and exercise *T*_core_ (Tables 3 and 4). There was a moderate,  but non-significant effect for a reduction in *T*_sk_ and a large non-significant effect for an increase in SR after a high TEE regime (Tables 3 and 4).

#### Frequency of Heat Exposure

There was no between-group difference for the frequency of heat adaptation regimes (consecutive/daily compared to non-consecutive/non-daily heat exposure) and a reduction in resting *T*_core_ (*p* = 0.18), exercise *T*_core_ (*p* = 0.99) and HR (*p* = 0.32) and an increase in SR (*p* = 0.37). Within-group analyses found that after a consecutive (daily) heat adaptation regime, there was a moderate effect for a reduction in resting *T*_core_, *T*_sk_ and HR and an increase in SR, coupled with a large effect for a reduction in exercise *T*_core_ (Tables 3 and 4). After a non-consecutive (non-daily) heat adaptation regime, there was a small effect for a reduction in HR and a large effect for a reduction in exercise *T*_core_ (Tables 3 and 4). There was a small non-significant effect for a reduction in resting *T*_core_, and a trivial effect for an increase in SR following a non-consecutive (non-daily) heat adaptation regime (Tables 3 and 4). There were insufficient studies (*k* = 1) to investigate *T*_sk_ changes for non-consecutive (non-daily) exposures.

#### Training Status

There was no between-group difference for training status (comparison of Tier 1 ‘Recreationally Active’ and Tier 2 ‘Trained/Developmental’) and a reduction in resting *T*_core_ (*p* = 0.61), *T*_sk_ (*p* = 0.21) and HR (*p* = 0.63). There was a between group difference for exercise *T*_core_ (*p* = 0.01), with a decrease in exercise *T*_core_ for females of Tier 2 but no change for Tier 1 classification (Table 3). Within Tier 1, there was a small effect for a reduction in resting *T*_core_ and a moderate effect for a decrease in *T*_sk_, and HR following heat adaptation (Tables 3 and 4). There was a trivial, but non-significant effect for a reduction in exercise *T*_core _ in females of Tier 1 status following heat adaptation (Table 3). There were insufficient studies (*k* = 1) to complete SR analyses for Tier 1. Within Tier 2, there was a moderate effect for a reduction in resting *T*_core_, and HR, and a large effect for a reduction in exercise *T*_core_, *T*_sk_ and an increase in SR following heat adaptation. Studies with participants of Tier 3 and Tier 4 calibre were included in this systematic review; however, they were not included in the meta-analysis, and therefore not investigated (*k* < 2).

#### Summary

**Fig. 9:**
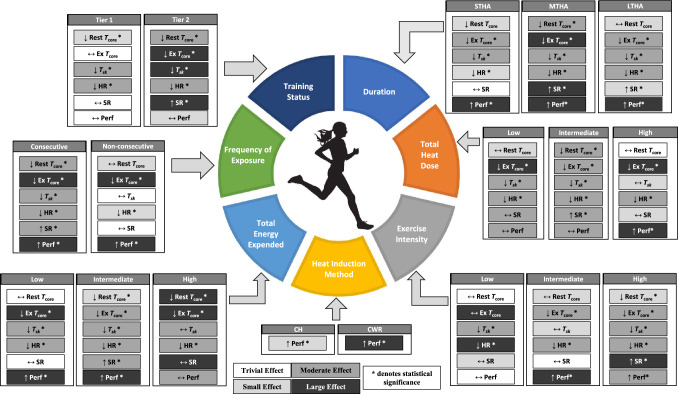
Summary figure of moderators on physiological adaptations and performance test outcomes in the heat following a heat adaptation regime. *CH* controlled hyperthermia, *CWR* controlled work rate, *Ex T*_core_ exercise core temperature, *HR* heart rate, *LTHA* long-term heat acclimation, *MTHA* medium-term heat acclimation, *Perf* exercise performance, *Rest T*_core_ resting core temperature, *SR* sweat rate, *STHA* short-term heat acclimation, *T*_sk_ skin temperature, ↑ indicates a beneficial increase following heat adaptation, ↓ indicates a beneficial decrease following heat adaptation, ↔ indicates no change after heat adaptation

A summary of the sub-group analyses for the physiological adaptations and performance test outcomes following heat adaptation in females is presented in Fig. 9.

### Meta-Regression

An exploratory meta-regression identified an inverse relationship between the magnitude of percentage change in performance test outcomes in the heat and a reduction in HR following heat adaptation regimes (standardised mean difference =  − 10 beats^.^min^−1^; 95% CI − 19, − 1; *r*^2^ = 0.72; *p* = 0.031). There was no association with the magnitude of percentage change in performance test outcomes in the heat and the other physiological variables following heat adaptation protocols. It should be noted that the analysis for all physiological variables and change in performance test outcomes should be considered as exploratory, given the limitations of this analysis with a small sample size (*k* < 10 studies).

## Discussion

The aim of this systematic review and meta-analysis was to examine the physiological adaptations and exercise performance outcomes in females following heat adaptation strategies. The key findings indicate that a reduction in resting and exercise *T*_core_, *T*_sk_ and HR, and an increase in SR occurs for females after exposure to heat adaptation regimes. There was no change in PV. Additionally, performance test outcomes in the heat are improved in females, with an increase in mean power and TTE following the use of heat adaptation strategies. Except for the heat induction method, where a between-group difference for controlled work rate and controlled hyperthermia was found, there were no other differences between the levels within each moderator for performance test outcomes in the heat for females. Within the levels of each moderator for the physiological adaptations to heat, a between-group difference for SR was found, with a greater increase in SR occurring in heat regimes utilising high EI compared with low EI. A between group difference for training status was also identified, with a reduction in exercise *T*_core_ occurring for females of Tier 2 but not Tier 1 classification following heat adaptation. The remaining  physiological changes to heat adaptation regimes were similar across the levels within each of the remaining moderators. Investigating within levels of each moderator revealed a more consistent pattern of statistically significant physiological change when the heat adaptation regime involved heat exposure durations of 451–900 min and/or 8–14 days, EI ≥ 3.5 kcal^.^min^−1^, TEE ≥ 3038 kcal, consecutive (daily) frequency and total heat dose ≥ 23,000 °C^.^min. Overall, these findings provide insight into the importance of various factors required to optimise the design of heat adaptation regimes to improve exercise performance and physiological adaptations in females.

### Physiological Adaptations

The meta-analysis clearly demonstrated that females physiologically responded to heat adaptation regimes with a reduction in resting and exercise *T*_core_, *T*_sk_ and HR and an increase in SR. These findings are similar to a meta-analysis with a predominantly male cohort [18] and suggest that at least at the macro level, females physiologically adapt to the heat in a similar manner as per their male counterparts. One major difference, however, was that this meta-analysis did not detect an increase in PV following a heat adaptation regime in females. It is unclear why this was the case. Possible reasons may include that this variable is regulated differently in females, that there was a lack of experimental control on the female menstrual cycle effects on fluid shifts masking any heat adaptation effects, and/or there was an inadequate number of studies available to meta-analyse in this review to enable detection of an increase in PV. The regulation of PV in females after heat adaptation regimes clearly remains an area requiring further research.

### Performance After Heat Adaptation

The meta-analysis demonstrated that performance test outcomes were improved following heat adaptation regimes in females. This is a novel result as this is the first meta-analysis to exclusively examine female performance in the heat following a heat adaptation regime. Irrespective of the duration of the heat adaptation regime (STHA, MTHA and LTHA), an improvement in performance test outcomes was identified. These findings are consistent with previous heat adaptation reviews in combined sex, majority male cohorts that also reported exercise performance improvements following heat adaptation regimes [18, 20]. Furthermore, performance test outcomes were also improved after controlled hyperthermia and controlled work rate methods, with the latter inducing larger percentage changes in performance. This finding is somewhat interesting considering the fundamental requirement of a heat adaptation regime to deliver repeated exposures to a heat stress of a sufficiently challenging and progressively increasing thermal impulse to induce physiological adaptations [17, 66, 67]. A controlled hyperthermia regime is often prescribed to fulfill such criteria, via attainment and maintenance of *T*_core_ (~ 38.5 °C). Controlled work rate protocols, typically prescribed via a set work rate for all heat exposures, are somewhat vulnerable to declining thermal stimuli over a heat adaptation regime. Whilst there was a greater representation of controlled work rate protocols within the studies included in this review, another possible explanation for this variation in outcome may relate to the menstrual cycle and its effect on body temperature. The elevated *T*_core_ in phase 4 of the female menstrual cycle, defined as + 7 days after ovulation [68], previously referred to as the mid-luteal phase, is reported to be maintained during exercise in the heat, albeit by a smaller magnitude, and does not appear to be mitigated by heat dissipation mechanisms [10]. When combined with the prescription of a controlled hyperthermia regime with a target temperature (~ 38.5 °C), a lower total thermal impulse is likely needed during phase 4 (mid-luteal phase) of the menstrual cycle to reach this *T*_core._ Although speculative, this type of heat adaptation protocol in females could result in a lower accumulated thermal heat stress and therefore heat adaptation compared with the controlled work rate protocol [13, 22]. This might explain the smaller performance test outcomes for such a heat adaptation regime. Future research is required to determine the influence of the menstrual cycle on overall thermal loads of a heat adaptation regime when prescribing controlled hyperthermia protocols and potential influence on performance in the heat.

### Sub-Group Analyses

#### Duration of Heat Adaptation

There were no differences between the various durations of heat exposure (STHA, MTHA, LTHA) for any of the physiological variables investigated in this review. This result suggests that irrespective of duration, similar beneficial thermoregulatory adaptations are induced in females and by implication, significant physiological adaptations are occurring during the early stages within a heat adaptation regime. In support of this conclusion, when we investigated within each level of duration (STHA, MTHA, LTHA), there were significant reductions in exercise *T*_core_, *T*_sk_ and HR after STHA. The only variables that did not change after STHA were resting *T*_core_ and SR, which were found to change after MTHA. These findings somewhat contrast with the summary provided by a recent narrative review of female heat adaptations [22], which concluded that an increase in SR occurred after STHA but decreases in exercise *T*_core_ and HR occurred only after MTHA [22]. Whilst the reasons for the conflicting findings are unclear, possible explanations for the divergent outcomes are that this review included data from mixed cohort studies without sex comparisons, whereas Wickham et al. [22] only included female-specific or sex-difference studies and only exercise-focused studies. Moreover, this review included studies with sedentary heat exposure only, for example, sauna and/or spa, whereas Wickham et al. [22] did not. Furthermore, additional studies have been published since the release of Wickham et al.’s review [22], which may have changed the outcome of the analyses.

Interestingly, our results identified a reduction in exercise *T*_core_ and *T*_sk_, without a corresponding increase in SR after STHA. The drop in temperatures (exercise *T*_core_ and *T*_*s*k_) during exercise with heat adaptation likely results from an increased capacity for heat loss [69]. The major heat loss mechanism during exercise in the heat is predominantly a function of evaporative heat loss [70]. Typically, some of the sweat produced during exercise in the heat is evaporated from the skin, contributing to body heat loss, whilst another portion of the sweat drips from the skin, which makes no contribution to body heat loss. The results of this meta-analysis would suggest that the increased heat loss response, represented by a reduction in exercise *T*_core_ and *T*_sk_ without an increase in SR after STHA, is due to a greater proportion of the same SR being evaporated. Future research therefore needs to identify whether heat loss via evaporative sweating increases without an increase in total SR in females following heat adaptation regimes of STHA. It should be noted that there was only a small sample size and number of studies available to include in the SR analysis in this review. Further investigations of the regulation of SR and its efficiency in females during heat adaptation regimes is therefore required.

#### Total Heat Dose of Heat Adaptation

The quantification of total heat dose is novel within heat adaptation research. The use of this term here is an attempt to provide an objective method of the quantification of heat exposure within a heat adaptation regime. Notably, the accumulation of total heat dose can be achieved in various ways including differing prescription methods such as exercise and non-exercise exposures, for example, saunas and hot water immersion, and acclimation or acclimatisation-based heat adaptation regimes. In this meta-analysis, there were no between-group differences for the level of heat dose (low, intermediate, high) on any of the analysed physiological adaptations induced after a heat adaptation regime. It can therefore be interpreted that irrespective of total heat dose, there are similar physiological adaptations arising following a heat adaptation regime. When investigating within total heat dose exposures, a significant reduction in exercise *T*_core_ and HR was identified with all total heat dose exposures, a significant reduction in *T*_sk_ with low and intermediate total heat dose, and a significant reduction in resting *T*_core_ and SR with an intermediate total heat dose only. The observation that the intermediate total heat dose was the only dose in which significant positive heat adaptations occurred for all of the physiological variables might suggest that this is the optimal total heat dose to induce beneficial thermoregulatory adaptations.

Interestingly, the beneficial physiological adaptations to heat might be attained irrespective of the method of exposure (e.g. sauna suit, mixed method or via exercise-based regime), if an intermediate total heat dose (23,001–43,200 °C^.^min) is undertaken. For example, when comparing three heat adaptation protocols for total heat dose: a non-exercise exposure (sauna, 7 days, 90 min at 40.5 °C; 25,515 °C^.^min) [50], mixed method of pre-exercise sauna suit (5 days, 20 min, 50 °C) followed by a controlled hyperthermia exercise regime (5 days, 90 min 40 °C; 23,000 °C^.^min) [36] and a controlled hyperthermia exercise regime (10 days, 60 min 40 °C, 24,000 °C^.^min) [60], a mean difference was observed for a reduction in resting and exercise *T*_core_, *T*_sk_ and HR and increase in SR. Whilst total heat dose can be accumulated in several ways, further research is required to understand if the induction method utilised in a heat adaptation regime to achieve a total heat dose influences the subsequent physiological adaptations. Additionally, there are other limitations to this interpretation. Specifically, it is important to note that all classifications of total heat dose (low, intermediate and high) were identified from the individual heat doses utilised by the studies in this review and therefore should be applied with caution. The importance of the use of total heat dose and its optimisation for heat adaptation protocols in females remains unknown and requires further study before it might be useful for athletes, coaches and applied sport practitioners.

#### Exercise Intensity Undertaken During Heat Adaptation

There was a between-group difference in SR with EI, whereby an increased SR was identified for heat adaptation regimes that employed high EI protocols compared with protocols utilising a low EI approach. For other physiological variables, however, there were no between-group differences, again suggesting a similar adaptation profile was achieved irrespective of the EI utilised in the heat adaptation regime for females. The increased SR identified when employing high EI protocols might be explained by a greater rate of metabolic heat production, driving an increased need for heat loss via an enhanced evaporative sweat loss to achieve heat balance [71]. When investigating within the EI levels, we found that most adaptations in the physiological variables occurred with intermediate-to-high EI but not low EI protocols. This suggests that heat adaptation regimes employing a higher EI (within reason) leads to more complete physiological adaptation to the heat in females.

#### Total Energy Expended During a Heat Adaptation

In the meta-analysis, there were no between-group differences for TEE (low, intermediate, high) on physiological adaptations induced after a heat adaptation regime. It can therefore be concluded that similar physiological adaptations arise irrespective of TEE in a heat adaptation regime. When examining within levels of TEE, it seems with intermediate TEE there is a greater number of statistically significant beneficial thermoregulatory adaptations induced in females after heat adaptation regimes. Unsurprisingly, given the method of TEE calculation in the current review (a product of EI and duration), most heat adaptation regimes with a high TEE were of MTHA and LTHA as a result of the extended duration of such protocols. Importantly however, thermoregulatory benefits of exercise in the heat, including a reduction in exercise *T*_core_, *T*_sk_ and HR, were reported after STHA and a high EI [37]. Such exposure is likely beneficial for females seeking to induce thermoregulatory adaptations beneficial for exercise in the heat with limited preparation time. It is therefore recommended that when developing a heat adaptation regime, consideration of the TEE is needed to ensure the protocol delivers the required stimulus. Future research focusing on heat adaptation protocols in females would benefit from continuing to quantify the TEE of heat regimes and subsequent physiological adaptations and performance outcomes to enhance knowledge of the usefulness and applicability of this variable in protocol prescription.

#### Frequency of Heat Adaptation

There were no between-group differences for the frequency of exposure (consecutive, non-consecutive) on physiological adaptations examined after a heat adaptation regime, which suggests that similar physiological adaptations arise in a heat adaptation regime, irrespective of the frequency of heat exposure. After investigation within levels, all physiological adaptations were identified to arise following a consecutive (daily) exposure. Whilst there were few studies included within this review that employed a non-consecutive protocol (*k* = 4) compared with a consecutive approach (*k* = 25), and a combined consecutive and non-consecutive approach (*k* = 1), there are practical outcomes that can be applied from the results of this meta-analysis. When deciding the frequency of heat exposure to utilise within a heat adaptation regime, the overall aim of the heat adaptation protocol, including the physiological adaptations to be induced, should first be considered. If achieving the most complete physiological adaptation is the focus of the heat adaptation protocol and/or seeking a rapid induction of thermoregulatory adaptations, a consecutive (daily) exposure would be recommended. A non-consecutive (non-daily) protocol may however be beneficial for an athlete with an already congested training schedule, where a restricted amount of time is able to be allocated to a heat adaptation regime. Finally, more research is required to determine if non-consecutive heat adaptation regimes can consistently induce resting *T*_core,_ *T*_sk_ and SR adaptations.

#### Training Status and Heat Adaptation

In the meta-analysis, there was a between group difference in training status (Tier 1, Tier 2) for a reduction in exercise *T*_core_, with a reduction occurring for females of Tier 2 but not Tier 1 classification. For other physiological adaptations, there were no between-group differences induced after a heat adaptation regime, suggesting that similar physiological adaptations arise in females irrespective of training status. When examining within levels of training status, females classified as Tier 2 achieved statistically significant thermoregulatory benefits for all physiological adaptations after heat adaptation regimes. When employing the framework used in this review [42] to previous research on males of Tier 2 calibre, they also reported beneficial physiological adaptations following a heat adaptation regime, including a decrease in exercise *T*_core_, *T*_sk_ and HR and an increase in SR [72]. In the current review, the interpretation that participants classified as Tier 1 achieve fewer physiological adaptations in the heat compared with Tier 2 should be treated with caution because of the smaller number of participants and larger variation in the data. Furthermore, analyses of the physiological adaptations in females classified in Tiers 3–5 was not conducted as there were insufficient studies (*k* < 2) able to be included in the current meta-analysis. Further research involving all the Tiers of training is required to better understand the relationship between training status and the magnitude of physiological adaptation to heat exposure in females.

### Meta-Regression

Finally, an exploratory meta-regression in the current review identified an association between a reduction in HR and an improvement in performance test outcomes in the heat after a heat adaptation regime. This relationship suggests that monitoring for reductions in HR during each session of a heat adaptation regime might be a useful biomarker not only for providing a measure of an athlete’s heat adaptation status prior to competing in the heat, but also indicating possible improvements in exercise performance in the heat. This finding should be taken with caution as this investigation was considered exploratory (*k* < 10 studies), with future research required to confirm this association and its possible practical application.

### Limitations

Across the 30 included studies in this systematic review and meta-analysis, a common limitation was the inconsistency in methods used to identify the female menstrual cycle phase and its possible influence on physiological adaptations and performance test outcomes in the heat. In the current review, seven out of 30 included studies reported scheduling performance and/or capacity testing to match a phase of the menstrual cycle. Given the small number of original investigations quantifying the female menstrual cycle, the influence of the female menstrual cycle on the time course of physiological adaptations to heat in this systematic review and meta-analysis is currently unknown. Whilst previous research has suggested the onset threshold, defined as the body temperature at onset of the effector response, occurs at higher temperatures for females than males [15, 73], the menstrual cycle was not reported or controlled for in these studies. The reported higher onset threshold for females to activate heat loss mechanisms may simply be an elevated *T*_core_ associated with phase 4 (mid-luteal phase) of the menstrual cycle [74–83]. When females are tested during phase 1 (early-follicular phase) of the menstrual cycle, there is little reported variation in onset temperature thresholds to commence heat loss responses between the sexes during exercise in the heat [11, 12]. Researchers undertaking future heat adaptation regimes investigating females are therefore encouraged to view current best practice guidelines [68] on how to control for the female menstrual cycle in sport and exercise research. In doing so, future mechanistic heat adaptation research investigating females can report menstrual cycle information alongside observed physiological changes and performance test outcomes in the heat.

There was a large variation in exercise type and performance tests within the studies included in this systematic review and meta-analysis. In heat adaptation sessions, running (continuous and intermittent), walking, and cycling or a combination of modes within a session were employed. Similarly, there was also a variation in exercise modes utilised in performance tests, including bench stepping, cycling, running, a combined cycling and running protocol, and walking. There was also a large variation in performance test outcomes including distance and/or time completed, mean and peak power, and physiological responses only to heat stress tests. This variability made it difficult to synthesize data to a common metric, with the representation of performance as a percentage change in this review possibly overstating the improvements in tests. Furthermore, whilst not considered in the analyses in this review, the period of time (minutes and/or days) between the final heat exposure and a performance test, for example, time trial, was varied within included studies. In the future, as the body of research grows, it is hoped separate categories, for example, TTE, peak power and time trial performance, could be investigated, rather than condensed into one category, for further understanding of performance test outcomes in the heat for females following heat adaptation regimes.

## Practical Applications

Based upon the within-level analyses from the current review, the following recommendations can be made when designing a heat adaptation regime for females:

- To achieve partial physiological adaptation to heat, the following may be prescribed. A heat adaptation regime of STHA exposure (≤ 450 min and/or ≤ 7 days; equivalent to ~ 65 min per day for 7 days), with low-to-high EI and TEE, completed via either a consecutive (daily) or non-consecutive (non-daily) frequency, with a total heat dose ≥ 23,000 °C^.^min.
- To achieve a more complete physiological adaptation to heat, the following may be prescribed. A heat adaptation regime of MTHA exposure (451–900 min and/or 8 − 14 days; equivalent to ~ 65 min per day for 14 days), with intermediate-to-high EI and TEE, and completed via a consecutive (daily) frequency, and a total heat dose ≥ 23,000 °C^.^min.
- Athletes and sport practitioners are encouraged to monitor for reductions in HR at regular intervals throughout the course of a heat adaptation regime for females. This is a practical, inexpensive, and non-invasive tool that might be utilised as a biomarker during heat adaptation regimes for females to identify possible improvements in exercise performance in the heat.

## Conclusions

This is the first systematic review and meta-analysis to exclusively examine physiological adaptations and performance test outcomes in females following heat adaptation regimes. These data add to the growing body of knowledge specific to females and heat adaptation. Performance test outcomes in the heat are improved following heat adaptation regimes. A reduction in resting and exercise *T*_core_, *T*_sk_ and HR, and an increase in SR arises with heat adaptation regimes for females, yet PV remained unchanged. Heat adaptation regimes being prescribed to females in order to achieve a more complete physiological adaptation are recommended to follow a framework of: MTHA exposure (451–900 min and/or 8 − 14 days; equivalent to ~ 65 min per day for 14 days), with exercise of intermediate-to-high EI and TEE, and completed via a consecutive (daily) frequency, and a total heat dose ≥ 23,000 °C^.^min. Future studies investigating the mechanisms of heat adaptation in females are recommended to account for the various phases of the female menstrual cycle in study designs and quantify the influence of the cycle on the time course of physiological adaptations to the heat.

## Supplementary Information

Below is the link to the electronic supplementary material.

Supplementary file1 (DOCX 23 KB)

Supplementary file2 (DOCX 18 KB)

Supplementary file3 (DOCX 54 KB)

Supplementary file4 (DOCX 346 KB)

Supplementary file5 (DOCX 775 KB)

Supplementary file6 (DOCX 32 KB)

Supplementary file7 (DOCX 270 KB)

## Declarations

### Availability of Data and Material

The data sets generated and/or analysed during the current study are available from the corresponding author on reasonable request.
