# Supplementary material for: Heat Adaptation for Females: A Systematic Review and Meta-Analysis of Physiological Adaptations and Exercise Performance in the Heat
Source: Sports Med. 2023 May 24;53(7):1395–421. doi: 10.1007/s40279-023-01831-2 (PMC10289939; doi:10.1007/s40279-023-01831-2)
Supplement: Supplementary file 2 — Supplementary file2 (DOCX 18 KB) [file 40279_2023_1831_MOESM2_ESM.docx]

**Online Resource S2**

**Title:** Heat Adaptation for Females: A Systematic Review and Meta-Analysis of Physiological Adaptations and Exercise Performance in the Heat.

**Journal**: Sports Medicine.

**Authors:** Monica K. Kelly^1^*, Steven J. Bowe^2,3^, William T. Jardine^1^, Dominique Condo^1^, Joshua H. Guy^4^, Rodney J. Snow^5^, and Amelia J. Carr^1^

^1^ Centre for Sport Research, Deakin University, 221 Burwood Highway, Burwood, VIC, 3125, Australia

^2^ Deakin Biostatistics Unit, Faculty of Health, Deakin University, 221 Burwood Highway, Burwood, VIC, 3125, Australia

^3^ Faculty and School of Health, Victoria University of Wellington, Kelburn Parade, Kelburn, Wellington, 6140, New Zealand

^4^ School of Health, Medical and Applied Sciences, Central Queensland University, Cairns, QLD, Australia

^5^ Institute for Physical Activity and Nutrition, Deakin University, 221 Burwood Highway, Burwood, VIC, 3125, Australia

**Corresponding author**: Monica Kelly ([monica.kelly@research.deakin.edu.au](mailto:monica.kelly@research.deakin.edu.au))

**Electronic Supplementary Material Appendix S2.** Example of a Search Strategy Conducted in Sport Discus (5/08/2020).

|  | **Limits applied** | |
| --- | --- | --- |
|  | Humans | |
|  | English language | |
| **Search number** | **Search terms** | **Number of results** |
| 1 | TI (Acclim* OR Acclimati?ation) OR AB (Acclim* OR Acclimati?ation) | 1,479 |
| 2 | DE Acclimatization OR “COLD adaptation” OR “Heat adaptation” | 1,127 |
| 3 | S1 OR S2 | 2,004 |
| 4 | TI (Female* OR Wom?n) OR AB (Female* OR Wom?n) | 145,504 |
| 5 | DE WOMEN athletes OR WOMEN professional athletes | 4,936 |
| 6 | S4 OR S5 | 147,177 |
| 7 | TI (Athlet* OR Physical* OR Exercis* OR Perform* OR Sport* OR “Team sport*” OR Compet*) OR AB (Athlet* OR Physical* OR Exercis* OR Perform* OR Sport* OR “Team sport*” OR Compet*) | 793,789 |
| 8 | DE Athletes OR DE Team sports OR DE Sports competitions OR DE Performance OR DE Exercise OR DE Endurance athletes OR DE Racing | 146,815 |
| 9 | S7 OR S8 | 830,504 |
| 10 | TI (Physiol* OR Adapt* OR “core temperature” OR “rectal temperature” OR “skin temperature” OR “heart rate” OR “oxygen consumption” OR “lactate concentration” OR “plasma volume” OR “sweat rate” OR “subjective measure*” OR “rating of perceived exertion” OR “thermal comfort” OR “thermal sensation” OR “work output” OR “menstrual cycle” OR “contraceptive pill” OR metabolism OR hormonal OR neuroendocrine OR adrenaline OR “heat illness” OR VO2max OR VO2peak OR “hemoglobin concentration” OR hematocrit OR “hemoglobin mass” OR “haemoglobin concentration” OR haematocrit OR “haemoglobin mass” OR VO2 OR “body mass” OR menstruation OR pregnancy OR amenorrhea) OR AB (Physiol* OR Adapt* OR “core temperature” OR “rectal temperature” OR “skin temperature” OR “heart rate” OR “oxygen consumption” OR “lactate concentration” OR “plasma volume” OR “sweat rate” OR “subjective measure*” OR “rating of perceived exertion” OR “thermal comfort” OR “thermal sensation” OR “work output” OR “menstrual cycle” OR “contraceptive pill” OR metabolism OR hormonal OR neuroendocrine OR adrenaline OR “heat illness” OR VO2max OR VO2peak OR “hemoglobin concentration” OR hematocrit OR “hemoglobin mass” OR “haemoglobin concentration” OR haematocrit OR “haemoglobin mass” OR VO2 OR “body mass” OR menstruation OR pregnancy OR amenorrhea) | 130,112 |
| 11 | TI (Heat OR Hot OR Cool OR Cold OR Tempor* OR Thermoneutral OR Thermo*) OR AB (Heat OR Hot OR Cool OR Cold OR Tempor* OR Thermoneutral OR Thermo*) | 44,779 |
| 12 | DE Heat OR DE “Heat adaptation” | 2,604 |
| 13 | S11 OR S12 | 45,409 |
| 14 | S3 AND S6 | 157 |
| 15 | S3 AND S6 AND S9 | 106 |
| 16 | S3 AND S6 AND S10 | 92 |
| 17 | S3 AND S6 AND S13 | 86 |
| 18 | S3 AND S6 AND S9 AND S10 AND S13 | 50 |
|  | **Total: 491 (with duplicates)** | |
