# Supplementary material for: Heat Adaptation for Females: A Systematic Review and Meta-Analysis of Physiological Adaptations and Exercise Performance in the Heat
Source: Sports Med. 2023 May 24;53(7):1395–421. doi: 10.1007/s40279-023-01831-2 (PMC10289939; doi:10.1007/s40279-023-01831-2)
Supplement: Supplementary file 3 — Supplementary file3 (DOCX 54 KB) [file 40279_2023_1831_MOESM3_ESM.docx]

**Online Resource S3**

**Title:** Heat Adaptation for Females: A Systematic Review and Meta-Analysis of Physiological Adaptations and Exercise Performance in the Heat.

**Journal**: Sports Medicine.

**Authors:** Monica K. Kelly^1^*, Steven J. Bowe^2,3^, William T. Jardine^1^, Dominique Condo^1^, Joshua H. Guy^4^, Rodney J. Snow^5^, and Amelia J. Carr^1^

^1^ Centre for Sport Research, Deakin University, 221 Burwood Highway, Burwood, VIC, 3125, Australia

^2^ Deakin Biostatistics Unit, Faculty of Health, Deakin University, 221 Burwood Highway, Burwood, VIC, 3125, Australia

^3^ Faculty and School of Health, Victoria University of Wellington, Kelburn Parade, Kelburn, Wellington, 6140, New Zealand

^4^ School of Health, Medical and Applied Sciences, Central Queensland University, Cairns, QLD, Australia

^5^ Institute for Physical Activity and Nutrition, Deakin University, 221 Burwood Highway, Burwood, VIC, 3125, Australia

**Corresponding author**: Monica Kelly ([monica.kelly@research.deakin.edu.au](mailto:monica.kelly@research.deakin.edu.au))

**Electronic Supplementary Material Appendix S3.**

**Table S1** A **s**ummary of the extracted data from included studies in this systematic review and meta-analysis

| **References** | **Time point of collection** | **Pre HA** | **Post HA** | **Performance/ capacity (% ∆)** | ***T*_core_ resting (°C ∆)** | ***T*_core_ exercise (°C ∆)** | ***T*_skin_ (°C ∆)** | **HR  (b.min^-1^ ∆)** | **SR (%∆)** | **PV  (%∆)** |
| --- | --- | --- | --- | --- | --- | --- | --- | --- | --- | --- |
| Alkemade et al. [1] | HST | Baseline | At end of 30 min | ✓ | ✓ | ✓ | ✓ | ✓ | ✓ | 🗶 |
| Avellini et al. [2] | HST | Baseline | 90 min – Equivalent time to baseline TTE and end of exp. | ✓ | ✓ | ✓ | ✓ | ✓ | ✓ | 🗶 |
| Barry et al. [3] ^a^ | First/Last | Baseline | End of Exp. | 🗶 | ✓ | 🗶 | ✓ | 🗶 | ✓ | 🗶 |
| Buono et al. [4] | First/Last | Baseline | End of exp. | 🗶 | 🗶 | ✓ | ✓ | ✓ | ✓ | 🗶 |
| Campbell et al. [5] | First/Last ExH only | Baseline | 30 min – Equivalent time to baseline | 🗶 | ✓ | ✓ | ✓ | ✓ | 🗶 | 🗶 |
| Cleland et al. [6] | First/Last | Baseline | End of exp. | ✓ | 🗶 | 🗶 | 🗶 | 🗶 | 🗶 | 🗶 |
| Cohen and Gisolfi [7] | HST | Baseline | End of exp. | ✓ | ✓ | ✓ | ✓ | ✓ | 🗶 | 🗶 |
| Fein et al. [8] ^c^ | First/Last |  | End of exp. | ✓  ✓ | 🗶  🗶 | 🗶  🗶 | 🗶  🗶 | 🗶  🗶 | 🗶  🗶 | 🗶  🗶 |
| Fein et al. [8] ^d^ | First/Last |  |  |  |  |  |  |  |  |  |
| Frye and Kamon [9] | HST | Baseline | 60 min - Equivalent time to baseline TTE and end of exp. | ✓ | 🗶 | ✓ | 🗶 | 🗶 | 🗶 | 🗶 |
| Garrett et al. [10] | HST | Baseline | End of exp | ✓ | 🗶 | ✓ | ✓ | 🗶 | 🗶 | 🗶 |
| Gore et al. [11] ^d e^ | NA |  |  | 🗶 | 🗶 | 🗶 | 🗶 | 🗶 | 🗶 | 🗶 |
| Greenleaf et al. [12] | First/Last | Baseline | End of exp. | 🗶 | 🗶 | 🗶 | 🗶 | ✓ | ✓ | ✓ |
| Henderson et al. [13] ^d^ | First/Last | Baseline | End of exp. | 🗶 | ✓ | 🗶 | 🗶 | ✓ | ✓ | 🗶 |
| Horstman and Christensen [14] | First/Last | Baseline | 50 min - Equivalent time to baseline and end of exp. | ✓ | 🗶 | ✓ | 🗶 | ✓ | 🗶 | 🗶 |
| Kampmann et al. [15] ^e^ | NA |  |  | 🗶 | 🗶 | 🗶 | 🗶 | 🗶 | 🗶 | 🗶 |
|  | NA |  |  | 🗶 | 🗶 | 🗶 | 🗶 | 🗶 | 🗶 | 🗶 |
|  | NA |  |  | 🗶 | 🗶 | 🗶 | 🗶 | 🗶 | 🗶 | 🗶 |
| Kirby et al. [16] | First/Last | Baseline | End of exp. | ✓ | ✓ | ✓ | ✓ | ✓ | ✓ | 🗶 |
| Kirby et al. [17] ^a^ | First/Last | Baseline | End of exp. | 🗶 | ✓ | ✓ | ✓ | ✓ | ✓ | ✓ |
| Mee et al. [18] | HST | Baseline | End of exp. | 🗶 | ✓ | ✓ | ✓ | ✓ | ✓ | 🗶 |
| Mee et al. [19] ^a^ | HST | Baseline | End of exp. | 🗶 | ✓ | ✓ | ✓ | ✓ | ✓ | 🗶 |
| Meylan et al. [20] ^d e^ | NA |  |  | 🗶 | 🗶 | 🗶 | 🗶 | 🗶 | 🗶 | 🗶 |
| Moss et al. [21] | HST | Baseline | End of exp. | 🗶 | ✓ | ✓ | ✓ | ✓ | ✓ | 🗶 |
| O’Toole et al. [22] | First/Last | Baseline | End of exp. | 🗶 | 🗶 | ✓ | 🗶 | ✓ | ✓ | 🗶 |
| Pethick et al. [23] ^e^ | NA |  |  | 🗶 | 🗶 | 🗶 | 🗶 | 🗶 | 🗶 | 🗶 |
| Philp et al. [24] ^e^ | NA |  |  | 🗶 | 🗶 | 🗶 | 🗶 | 🗶 | 🗶 | 🗶 |
| Sawka et al. [25] (HA) ^e^ | NA |  |  | 🗶 | 🗶 | 🗶 | 🗶 | 🗶 | 🗶 | 🗶 |
| Sawka et al. [26] (Hypo – Hot-dry) | HST | Baseline | End of exp. | 🗶 | 🗶 | ✓ | ✓ | ✓ | ✓ | 🗶 |
| Sawka et al. [26] (Hypo – Hot-humid)^e^ | NA |  |  | 🗶 | 🗶 | 🗶 | 🗶 | 🗶 | 🗶 | 🗶 |
| Shapiro et al. [27] | First/Last | Baseline | End of exp. | 🗶 | 🗶 | ✓ | ✓ | ✓ | ✓ | 🗶 |
| Stephenson et al. [28] ^a^ | HST | Baseline | End of exp. | ✓ | ✓ | ✓ | ✓ | ✓ | ✓ | 🗶 |
| Sunderland et al. [29] | HST | Baseline | 38 min and/or end of set 2 – Equivalent time to baseline and end of exp. | ✓ | ✓ | ✓ | 🗶 | ✓ | 🗶 | ✓ |
| Wyndham et al. [30] ^e^ | NA |  |  | 🗶 | 🗶 | 🗶 | 🗶 | 🗶 | 🗶 | 🗶 |

*Key*: ^a^, includes passive heat exposure; ^b^, consecutive; ^c^, non-consecutive; ^d^, acclimatisation; ^e^, included in the systematic review only; ExH, exercise in the heat; Exp, exposure; HA heat adaptation; HR, heart rate; HST, heat stress test; Min, minutes; Perf, performance; PV, plasma volume; SR, sweat rate; *T*_core_, core temperature; *T*_sk_, skin temperature; TTE, time to exhaustion.

**Reference List:**

1. Alkemade, P., et al. Individual characteristics associated with the magnitude of heat acclimation adaptations*.* Eur J Appl Physiol. 2021;1216:1593-1606; <https://10.1007/s00421-021-04626-3>.

2. Avellini, B.A., E. Kamon, and J.T. Krajewski. Physiological responses of physically fit men and women to acclimation to humid heat*.* J Appl Physiol Respir Environ Exerc Physiol. 1980;492:254-61; <https://10.1152/jappl.1980.49.2.254>.

3. Barry, H., et al. Improved neural control of body temperature following heat acclimation in humans*.* J Physiol. 2020;5986:1223-1234; <https://10.1113/JP279266>.

4. Buono, M.J., S. Leichliter Martha, and J.H. Heaney. Peripheral sweat gland function, but not whole-body sweat rate, increases in women following humid heat acclimation*.* J Therm Biol. 2010;353:134-137; <https://10.1016/j.jtherbio.2010.01.004>.

5. Campbell, H.A., et al. Acute physiological and psychophysical responses to different modes of heat stress*.* Exp Physiol. 2022;1075:429-440; <https://10.1113/ep089992>.

6. Cleland, T.S., S.M. Horvath, and M. Phillips. Acclimatization of women to heat after training*.* Int Z Angew Physiol. 1969;271:15-24; <https://10.1007/BF00695014>.

7. Cohen, J.S. and C.V. Gisolfi. Effects of interval training on work-heat tolerance of young women*.* Med Sci Sports Exerc. 1982;141:46-52; <https://10.1249/00005768-198201000-00009>.

8. Fein, J.T., E.M. Haymes, and E.R. Buskirk. Effects of daily and intermittent exposures on heat acclimation of women*.* Int J Biometeorol. 1975;191:41-52; <https://10.1007/BF01459840>.

9. Frye, A.J. and E. Kamon. Responses to dry heat of men and women with similar aerobic capacities*.* J Appl Physiol Respir Environ Exerc Physiol. 1981;501:65-70; <https://https://doi.org/10.1152/jappl.1981.50.1.65>.

10. Garrett, A.T., et al. Effectiveness of short-term heat acclimation on intermittent sprint performance with moderately trained females controlling for menstrual cycle phase*.* Front Physiol. 2019;10:1458; <https://10.3389/fphys.2019.01458>.

11. Gore, C.J., et al. VO2max and haemoglobin mass of trained athletes during high intensity training*.* Int J Sports Med. 1997;186:477-482; <https://DOI>: 10.1055/s-2007-972667.

12. Greenleaf, J.E., P.J. Brock, and D. Sciaraffa. Effects of exercise-heat acclimation on fluid, electrolyte, and endocrine responses during tilt and +Gz acceleration in women and men*.* Aviat Space Environ Med. 1985;567:683-689.

13. Henderson, M.J., et al. Responses to a 5-day sport-specific heat acclimatization camp in elite female rugby sevens athletes*.* Int J Sports Physiol Perform. 2022:1-10; <https://10.1123/ijspp.2021-0406>.

14. Horstman, D.H. and E. Christensen. Acclimatization to dry heat: active men vs. active women*.* J Appl Physiol Respir Environ Exerc Physiol. 1982;524:825-31; <https://10.1152/jappl.1982.52.4.825>.

15. Kampmann, B., et al. Lowering of resting core temperature during acclimation is influenced by exercise stimulus*.* Euro J Appl Physiol. 2008;1042:321-327; <https://10.1007/s00421-007-0658-6>.

16. Kirby, N.V., S.J.E. Lucas, and R.A.I. Lucas. Nine-, but not four-days heat acclimation improves self-paced endurance performance in females*.* Front Physiol. 2019;10MAY:539; <https://10.3389/fphys.2019.00539>.

17. Kirby, N.V., et al. Sex differences in adaptation to intermittent post-exercise sauna bathing in trained middle-distance runners*.* Sports Med Open. 2021;71:51; <https://10.1186/s40798-021-00342-6>.

18. Mee, J.A., et al. A comparison of males and females' temporal patterning to short- and long-term heat acclimation*.* Scand J Med Sci Sports. 2015;25 Suppl 1:250-8; <https://10.1111/sms.12417>.

19. Mee, J.A., et al. Sauna exposure immediately prior to short-term heat acclimation accelerates phenotypic adaptation in females*.* J Sci Med Sport. 2018;212:190-195; <https://10.1016/j.jsams.2017.06.024>.

20. Meylan, C.M., et al. The efficacy of heat acclimatization pre-world cup in female soccer players*.* Front Sports Act Living. 2021;3:116; <https://https://doi.org/10.3389/fspor.2021.614370>.

21. Moss, J.N., et al. Short-term isothermic heat acclimation elicits beneficial adaptations but medium-term elicits a more complete adaptation*.* Euro J Appl Physiol. 2020;1201:243-254; <https://10.1007/s00421-019-04269-5>.

22. O'Toole, M.L., et al. The effects of heat acclimation on plasma volume and plasma protein of females*.* Int J Sports Med. 1983;41:40-4; <https://10.1055/s-2008-1026014>.

23. Pethick, W.A., et al. The effect of a team sport-specific heat acclimation protocol on plasma volume in elite female soccer players*.* Sci Med Footb. 2018;21:16-22; <https://10.1080/24733938.2017.1384559>.

24. Philp, C.P., et al. Can ten days of heat acclimation training improve temperate-condition rowing performance in national-level rowers? PloS one. 2022;179:e0273909; <https://10.1371/journal.pone.0273909>.

25. Sawka, M.N., et al. Does heat acclimation lower the rate of metabolism elicited by muscular exercise? Aviat Space Environ Med. 1983;541:27-31.

26. Sawka, M.N., et al. Hypohydration and exercise: effects of heat acclimation, gender, and environment*.* J Appl Physiol Respir Environ Exerc Physiol. 1983;554:1147-53; <https://10.1152/jappl.1983.55.4.1147>.

27. Shapiro, Y., K.B. Pandolf, and R.F. Goldman. Sex differences in acclimation to a hot-dry environment*.* Ergonomics. 1980;237:635-42; <https://10.1080/00140138008924778>.

28. Stephenson, B.T., K. Tolfrey, and V.L. Goosey-Tolfrey. Mixed active and passive, heart rate-controlled heat acclimation is effective for paralympic and able-bodied triathletes*.* Front Physiol. 2019;10:1214; <https://https://doi.org/10.3389/fphys.2019.01214>.

29. Sunderland, C., J.G. Morris, and M.E. Nevill. A heat acclimation protocol for team sports*.* Br J Sports Med. 2008;425:327-33; <https://10.1136/bjsm.2007.034207>.

30. Wyndham, C.H., J.F. Morrison, and C.G. Williams. Heat reactions of male and female Caucasians*.* J Appl Physiol. 1965;203:357-64; <https://10.1152/jappl.1965.20.3.357>.
