# Supplementary material for: Heat Adaptation for Females: A Systematic Review and Meta-Analysis of Physiological Adaptations and Exercise Performance in the Heat
Source: Sports Med. 2023 May 24;53(7):1395–421. doi: 10.1007/s40279-023-01831-2 (PMC10289939; doi:10.1007/s40279-023-01831-2)
Supplement: Supplementary file 4 — Supplementary file4 (DOCX 346 KB) [file 40279_2023_1831_MOESM4_ESM.docx]

**Online Resource 4**

**Title:** Heat Adaptation for Females: A Systematic Review and Meta-Analysis of Physiological Adaptations and Exercise Performance in the Heat.

**Journal:** Sports Medicine.

**Authors:** Monica K. Kelly^1^*, Steven J. Bowe^2,3^, William T. Jardine^1^, Dominique Condo^1^, Joshua H. Guy^4^, Rodney J. Snow^5^, and Amelia J. Carr^1^

^1^ Centre for Sport Research, Deakin University, 221 Burwood Highway, Burwood, VIC, 3125, Australia

^2^ Deakin Biostatistics Unit, Faculty of Health, Deakin University, 221 Burwood Highway, Burwood, VIC, 3125, Australia

^3^ Faculty and School of Health, Victoria University of Wellington, Kelburn Parade, Kelburn, Wellington, 6140, New Zealand

^4^ School of Health, Medical and Applied Sciences, Central Queensland University, Cairns, QLD, Australia

^5^ Institute for Physical Activity and Nutrition, Deakin University, 221 Burwood Highway, Burwood, VIC, 3125, Australia

**Corresponding author:** Monica Kelly ([monica.kelly@research.deakin.edu.au](mailto:monica.kelly@research.deakin.edu.au))

**Electronic Supplementary Material Appendix S4.** Risk of Bias


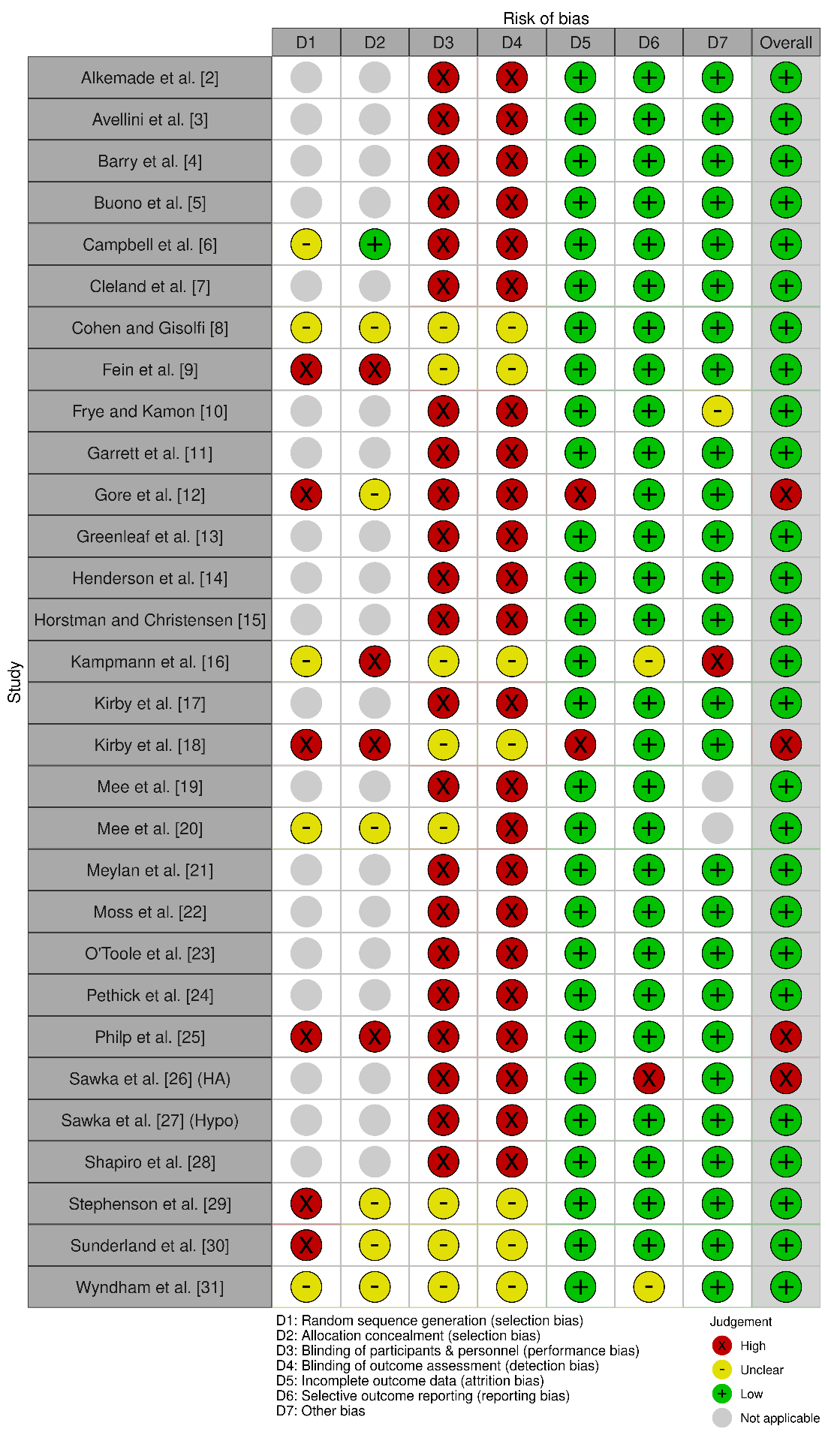

**Fig. S1** Risk of Bias for included studies in this systematic review and meta-analysis. This figure was produced using Robvis tool [1]

**Reference List:**

1. McGuinness, L.A. and J.P.T. Higgins. Risk-of-bias VISualization (robvis): An R package and Shiny web app for visualizing risk-of-bias assessments. Research Synthesis Methods. 2020;n/an/a https://10.1002/jrsm.1411.

2. Alkemade, P., et al. Individual characteristics associated with the magnitude of heat acclimation adaptations. Eur J Appl Physiol. 2021;1216:1593-1606; https://10.1007/s00421-021-04626-3.

3. Avellini, B.A., E. Kamon, and J.T. Krajewski. Physiological responses of physically fit men and women to acclimation to humid heat. J Appl Physiol Respir Environ Exerc Physiol. 1980;492:254-61; https://10.1152/jappl.1980.49.2.254.

4. Barry, H., et al. Improved neural control of body temperature following heat acclimation in humans. J Physiol. 2020;5986:1223-1234; https://10.1113/JP279266.

5. Buono, M.J., S. Leichliter Martha, and J.H. Heaney. Peripheral sweat gland function, but not whole-body sweat rate, increases in women following humid heat acclimation. J Therm Biol. 2010;353:134-137; https://10.1016/j.jtherbio.2010.01.004.

6. Campbell, H.A., et al. Acute physiological and psychophysical responses to different modes of heat stress. Exp Physiol. 2022;1075:429-440; https://10.1113/ep089992.

7. Cleland, T.S., S.M. Horvath, and M. Phillips. Acclimatization of women to heat after training. Int Z Angew Physiol. 1969;271:15-24; https://10.1007/BF00695014.

8. Cohen, J.S. and C.V. Gisolfi. Effects of interval training on work-heat tolerance of young women. Med Sci Sports Exerc. 1982;141:46-52; https://10.1249/00005768-198201000-00009.

9. Fein, J.T., E.M. Haymes, and E.R. Buskirk. Effects of daily and intermittent exposures on heat acclimation of women. Int J Biometeorol. 1975;191:41-52; https://10.1007/BF01459840.

10. Frye, A.J. and E. Kamon. Responses to dry heat of men and women with similar aerobic capacities. J Appl Physiol Respir Environ Exerc Physiol. 1981;501:65-70; https://https://doi.org/10.1152/jappl.1981.50.1.65.

11. Garrett, A.T., et al. Effectiveness of short-term heat acclimation on intermittent sprint performance with moderately trained females controlling for menstrual cycle phase. Front Physiol. 2019;10:1458; https://10.3389/fphys.2019.01458.

12. Gore, C.J., et al. VO2max and haemoglobin mass of trained athletes during high intensity training. Int J Sports Med. 1997;186:477-482; https://DOI: 10.1055/s-2007-972667.

13. Greenleaf, J.E., P.J. Brock, and D. Sciaraffa. Effects of exercise-heat acclimation on fluid, electrolyte, and endocrine responses during tilt and +Gz acceleration in women and men. Aviat Space Environ Med. 1985;567:683-689.

14. Henderson, M.J., et al. Responses to a 5-day sport-specific heat acclimatization camp in elite female rugby sevens athletes. Int J Sports Physiol Perform. 2022:1-10; https://10.1123/ijspp.2021-0406.

15. Horstman, D.H. and E. Christensen. Acclimatization to dry heat: active men vs. active women. J Appl Physiol Respir Environ Exerc Physiol. 1982;524:825-31; https://10.1152/jappl.1982.52.4.825.

16. Kampmann, B., et al. Lowering of resting core temperature during acclimation is influenced by exercise stimulus. Euro J Appl Physiol. 2008;1042:321-327; https://10.1007/s00421-007-0658-6.

17. Kirby, N.V., S.J.E. Lucas, and R.A.I. Lucas. Nine-, but not four-days heat acclimation improves self-paced endurance performance in females. Front Physiol. 2019;10MAY:539; https://10.3389/fphys.2019.00539.

18. Kirby, N.V., et al. Sex differences in adaptation to intermittent post-exercise sauna bathing in trained middle-distance runners. Sports Med Open. 2021;71:51; https://10.1186/s40798-021-00342-6.

19. Mee, J.A., et al. A comparison of males and females' temporal patterning to short- and long-term heat acclimation. Scand J Med Sci Sports. 2015;25 Suppl 1:250-8; https://10.1111/sms.12417.

20. Mee, J.A., et al. Sauna exposure immediately prior to short-term heat acclimation accelerates phenotypic adaptation in females. J Sci Med Sport. 2018;212:190-195; https://10.1016/j.jsams.2017.06.024.

21. Meylan, C.M., et al. The efficacy of heat acclimatization pre-world cup in female soccer players. Front Sports Act Living. 2021;3:116; https://https://doi.org/10.3389/fspor.2021.614370.

22. Moss, J.N., et al. Short-term isothermic heat acclimation elicits beneficial adaptations but medium-term elicits a more complete adaptation. Euro J Appl Physiol. 2020;1201:243-254; https://10.1007/s00421-019-04269-5.

23. O'Toole, M.L., et al. The effects of heat acclimation on plasma volume and plasma protein of females. Int J Sports Med. 1983;41:40-4; https://10.1055/s-2008-1026014.

24. Pethick, W.A., et al. The effect of a team sport-specific heat acclimation protocol on plasma volume in elite female soccer players. Sci Med Footb. 2018;21:16-22; https://10.1080/24733938.2017.1384559.

25. Philp, C.P., et al. Can ten days of heat acclimation training improve temperate-condition rowing performance in national-level rowers? PloS one. 2022;179:e0273909; https://10.1371/journal.pone.0273909.

26. Sawka, M.N., et al. Does heat acclimation lower the rate of metabolism elicited by muscular exercise? Aviat Space Environ Med. 1983;541:27-31.

27. Sawka, M.N., et al. Hypohydration and exercise: effects of heat acclimation, gender, and environment. J Appl Physiol Respir Environ Exerc Physiol. 1983;554:1147-53; https://10.1152/jappl.1983.55.4.1147.

28. Shapiro, Y., K.B. Pandolf, and R.F. Goldman. Sex differences in acclimation to a hot-dry environment. Ergonomics. 1980;237:635-42; https://10.1080/00140138008924778.

29. Stephenson, B.T., K. Tolfrey, and V.L. Goosey-Tolfrey. Mixed active and passive, heart rate-controlled heat acclimation is effective for paralympic and able-bodied triathletes. Front Physiol. 2019;10:1214; https://https://doi.org/10.3389/fphys.2019.01214.

30. Sunderland, C., J.G. Morris, and M.E. Nevill. A heat acclimation protocol for team sports. Br J Sports Med. 2008;425:327-33; https://10.1136/bjsm.2007.034207.

31. Wyndham, C.H., J.F. Morrison, and C.G. Williams. Heat reactions of male and female Caucasians. J Appl Physiol. 1965;203:357-64; https://10.1152/jappl.1965.20.3.357.
