# Supplementary material for: Heat Adaptation for Females: A Systematic Review and Meta-Analysis of Physiological Adaptations and Exercise Performance in the Heat
Source: Sports Med. 2023 May 24;53(7):1395–421. doi: 10.1007/s40279-023-01831-2 (PMC10289939; doi:10.1007/s40279-023-01831-2)
Supplement: Supplementary file 5 — Supplementary file5 (DOCX 775 KB) [file 40279_2023_1831_MOESM5_ESM.docx]

**Online Resource 5**

**Title:** Heat Adaptation for Females: A Systematic Review and Meta-Analysis of Physiological Adaptations and Exercise Performance in the Heat.

**Journal**: Sports Medicine.

**Authors:** Monica K. Kelly^1^*, Steven J. Bowe^2,3^, William T. Jardine^1^, Dominique Condo^1^, Joshua H. Guy^4^, Rodney J. Snow^5^, and Amelia J. Carr^1^

^1^ Centre for Sport Research, Deakin University, 221 Burwood Highway, Burwood, VIC, 3125, Australia

^2^ Deakin Biostatistics Unit, Faculty of Health, Deakin University, 221 Burwood Highway, Burwood, VIC, 3125, Australia

^3^ Faculty and School of Health, Victoria University of Wellington, Kelburn Parade, Kelburn, Wellington, 6140, New Zealand

^4^ School of Health, Medical and Applied Sciences, Central Queensland University, Cairns, QLD, Australia

^5^ Institute for Physical Activity and Nutrition, Deakin University, 221 Burwood Highway, Burwood, VIC, 3125, Australia

**Corresponding author**: Monica Kelly ([monica.kelly@research.deakin.edu.au](mailto:monica.kelly@research.deakin.edu.au))

**Electronic Supplementary Material Appendix S5.** Risk of bias plots for publication bias

**
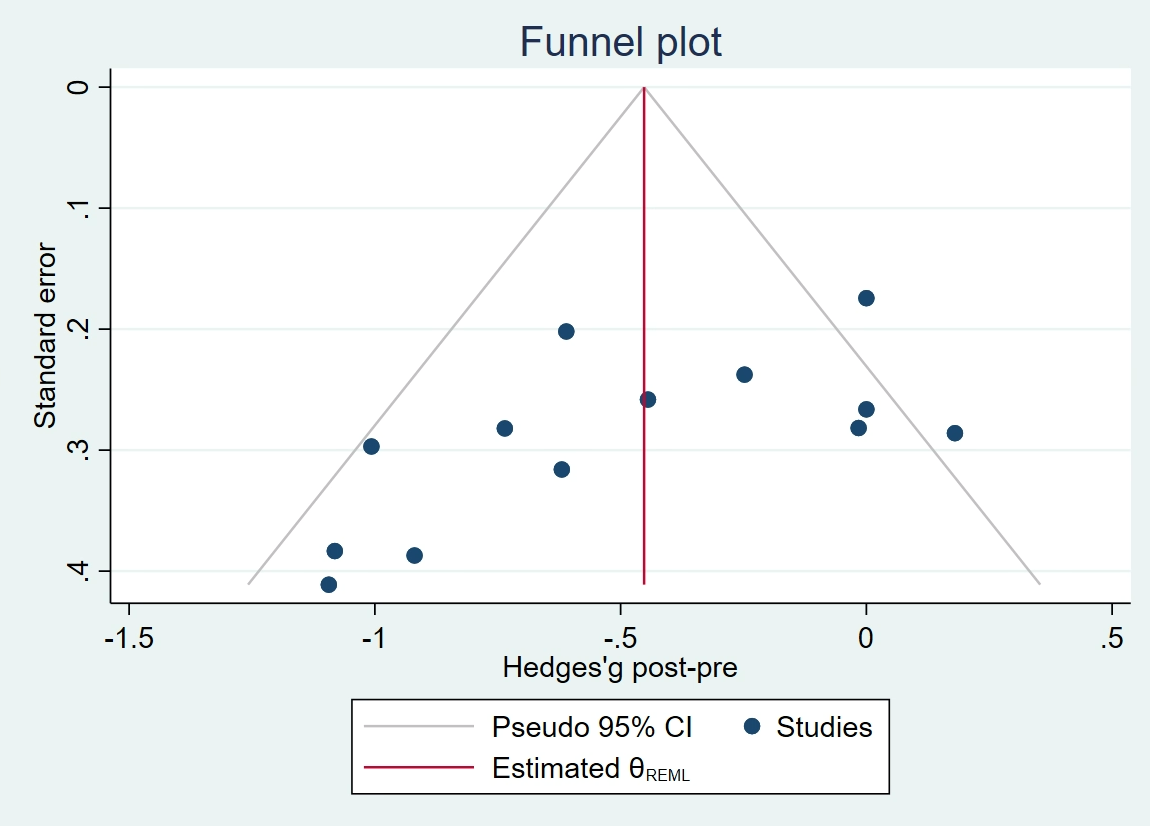
Fig. S1** Risk of bias plot for measures of resting core temperature


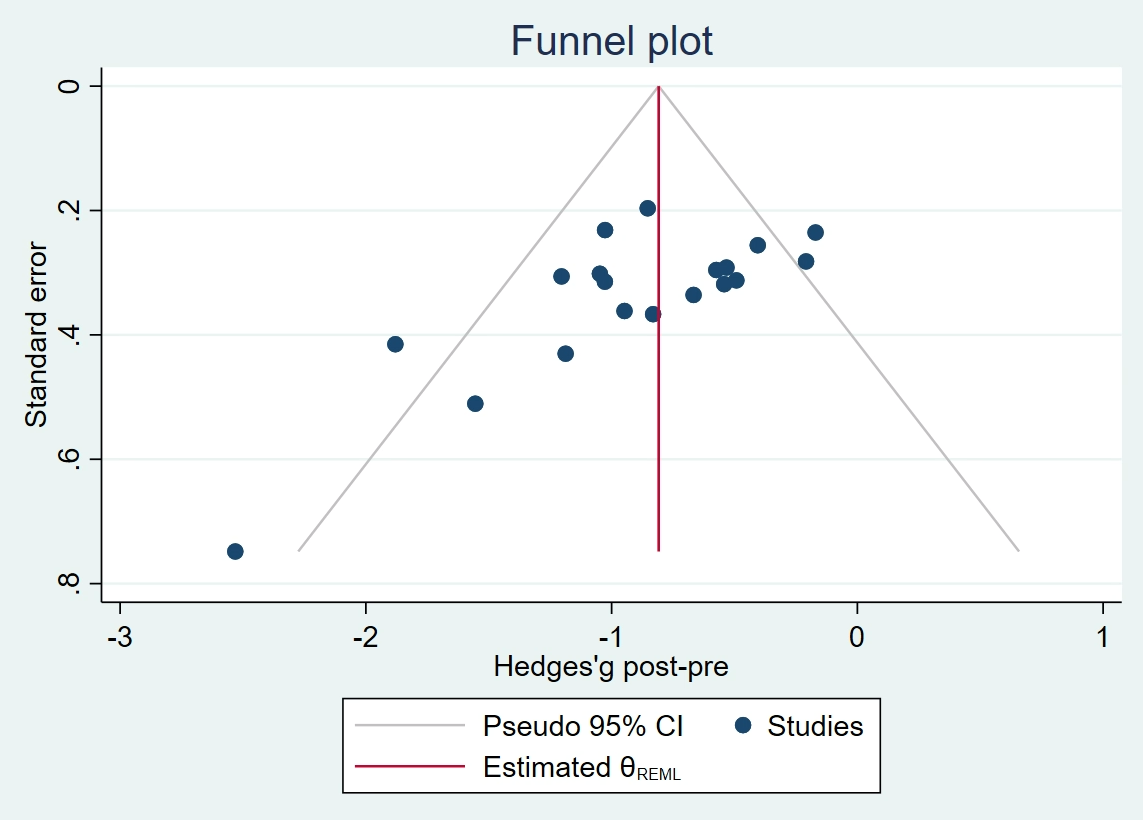


**Fig. S2** Risk of bias plot for measures of exercise core temperature


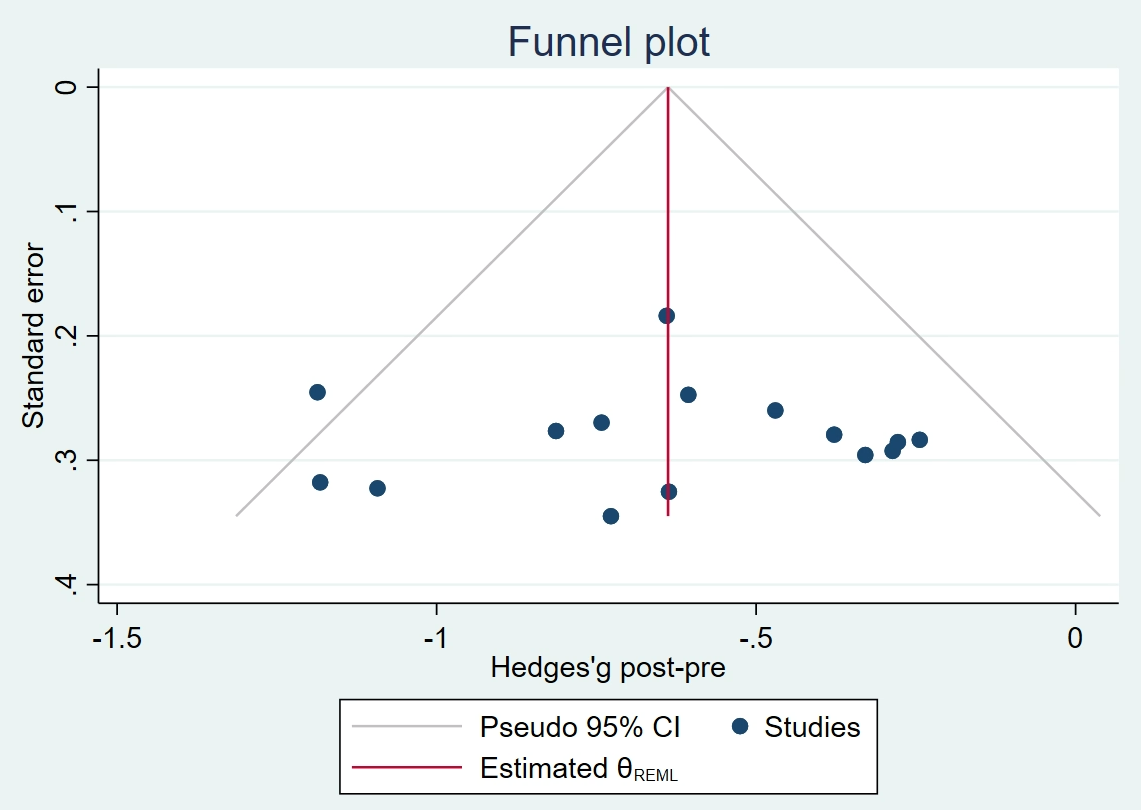


**Fig. S3** Risk of bias plot for measures of skin temperature


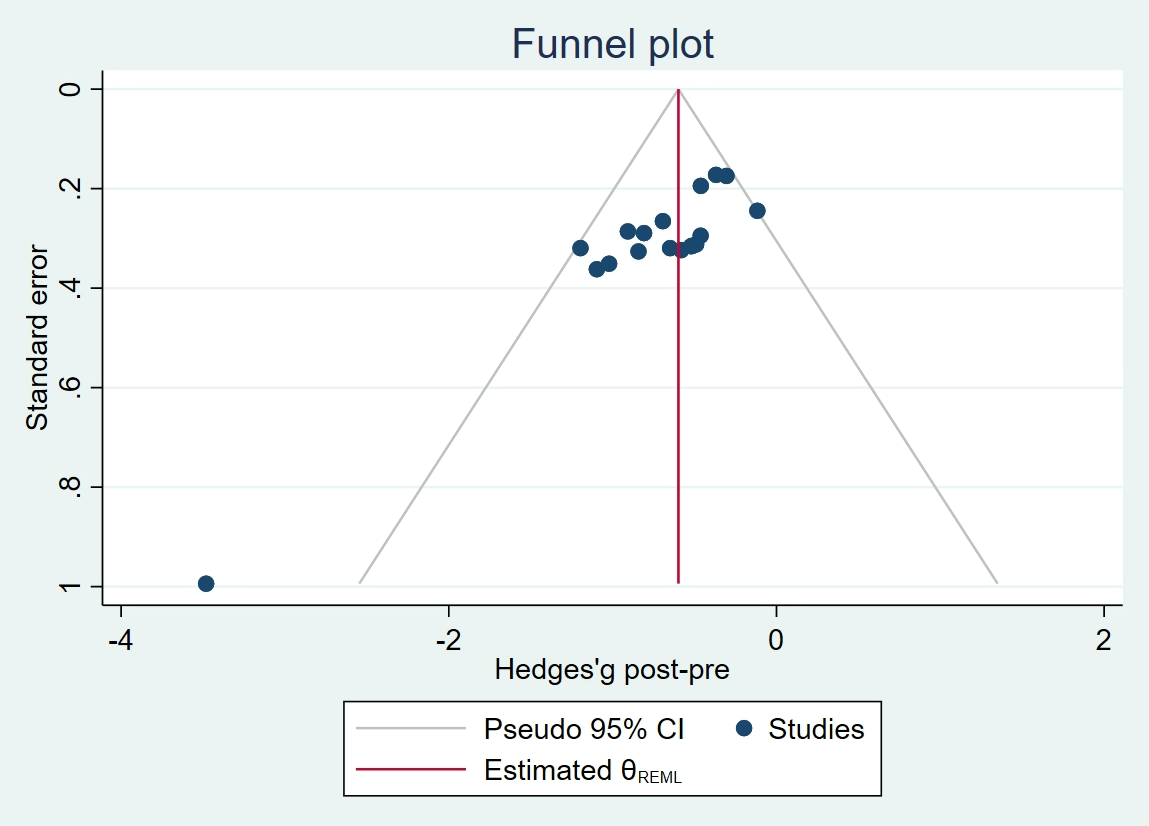


**Fig. S4** Risk of bias plot for measures of heart rate


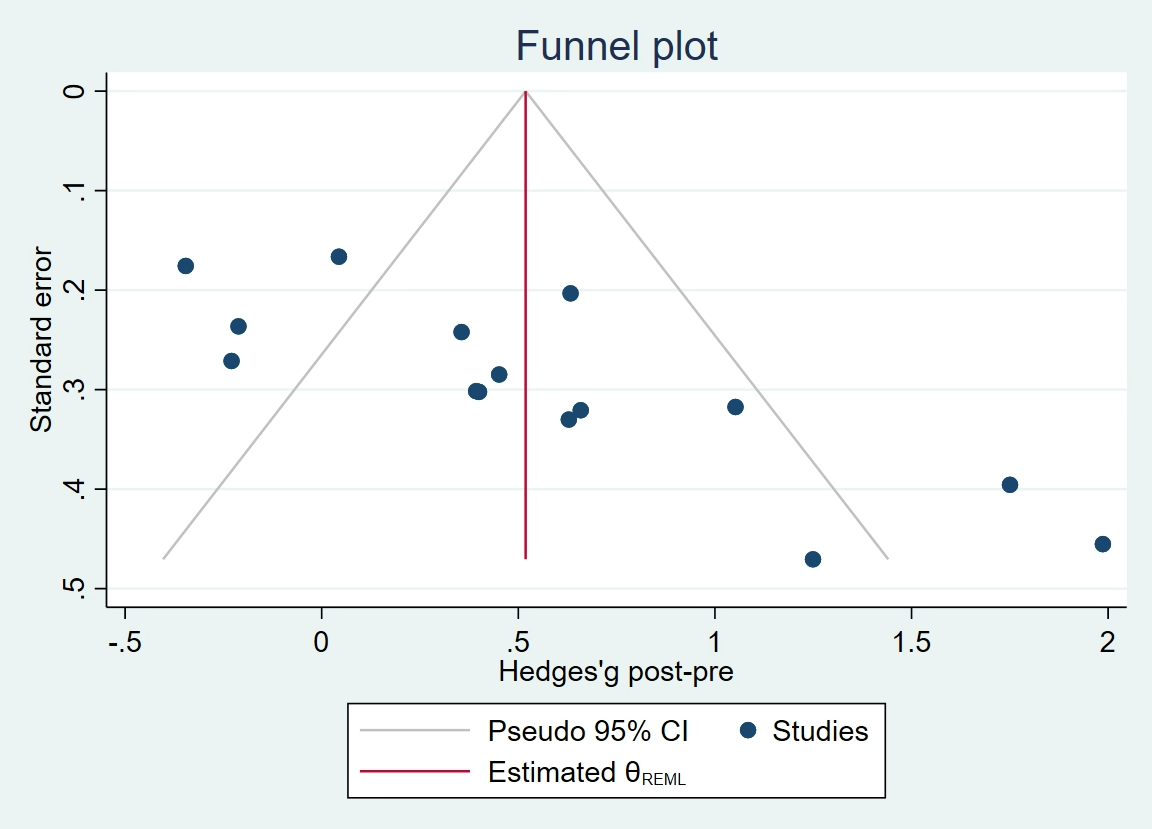


**Fig. S5** Risk of bias plot for measures of sweat rate


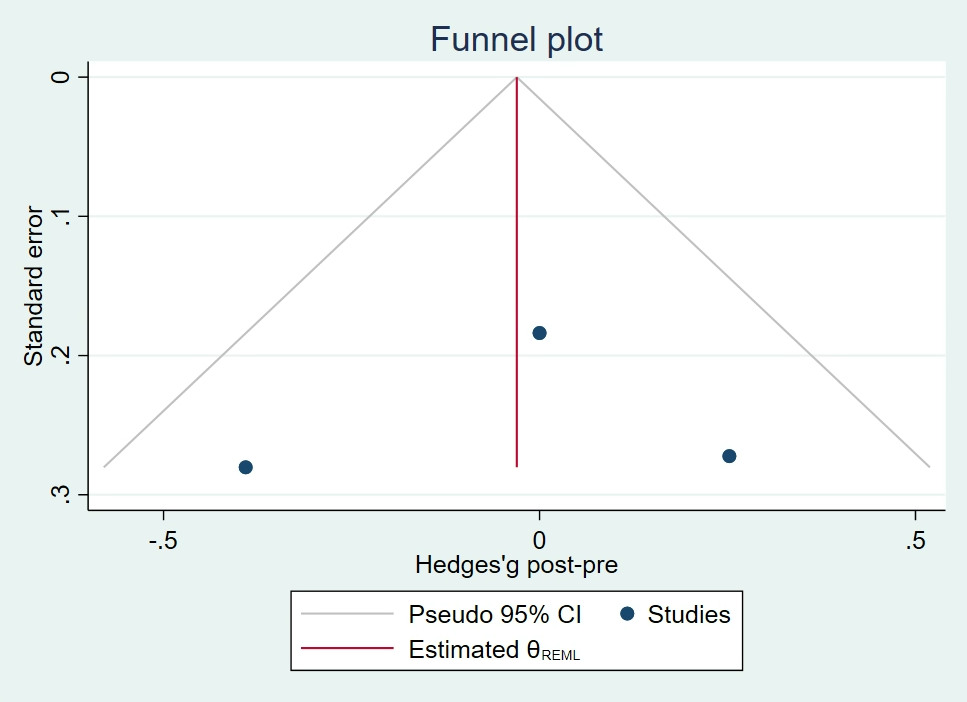


**Fig. S6** Risk of bias plot for measures of plasma volume

**
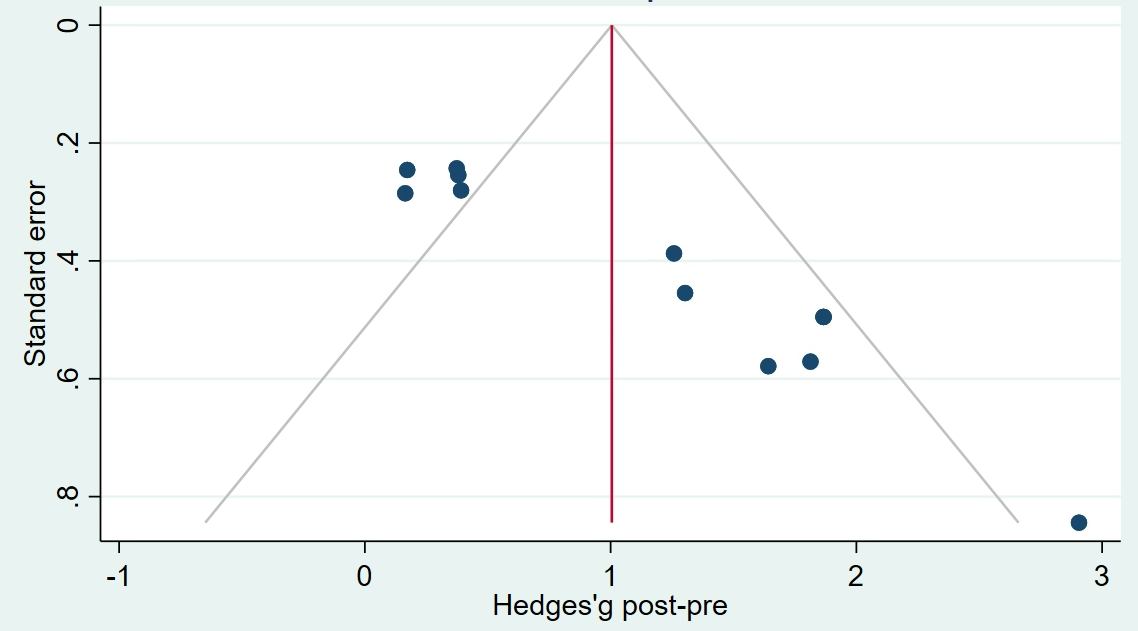
**

**Fig. S7** Risk of bias plot for performance
