# Supplementary material for: Heat Adaptation for Females: A Systematic Review and Meta-Analysis of Physiological Adaptations and Exercise Performance in the Heat
Source: Sports Med. 2023 May 24;53(7):1395–421. doi: 10.1007/s40279-023-01831-2 (PMC10289939; doi:10.1007/s40279-023-01831-2)
Supplement: Supplementary file 6 — Supplementary file6 (DOCX 32 KB) [file 40279_2023_1831_MOESM6_ESM.docx]

**Online Resource 6**

**Title:** Heat Adaptation for Females: A Systematic Review and Meta-Analysis of Physiological Adaptations and Exercise Performance in the Heat.

**Journal**: Sports Medicine.

**Authors:** Monica K. Kelly^1^*, Steven J. Bowe^2,3^, William T. Jardine^1^, Dominique Condo^1^, Joshua H. Guy^4^, Rodney J. Snow^5^, and Amelia J. Carr^1^

^1^ Centre for Sport Research, Deakin University, 221 Burwood Highway, Burwood, VIC, 3125, Australia

^2^ Deakin Biostatistics Unit, Faculty of Health, Deakin University, 221 Burwood Highway, Burwood, VIC, 3125, Australia

^3^ Faculty and School of Health, Victoria University of Wellington, Kelburn Parade, Kelburn, Wellington, 6140, New Zealand

^4^ School of Health, Medical and Applied Sciences, Central Queensland University, Cairns, QLD, Australia

^5^ Institute for Physical Activity and Nutrition, Deakin University, 221 Burwood Highway, Burwood, VIC, 3125, Australia

**Corresponding author**: Monica Kelly ([monica.kelly@research.deakin.edu.au](mailto:monica.kelly@research.deakin.edu.au))

**Electronic Supplementary Material Appendix S6.** Characteristics of included studies

**Table S1** Characteristics of included studies

| **Study** | **Total sample (n)** | **Sample (n) meta-analysed** | **Age**  **(year)** | **V̇O_2_max (ml.kg^-1^.min^-1^)** | **Athlete calibre** | **Menstrual phase information** | **Time between final heat exposure and physiological/ performance tests** |
| --- | --- | --- | --- | --- | --- | --- | --- |
| Alkemade et al. [1] | F 9  M 15 | F 9 | 31.3 (±7.4) | 45.9 (±6.4) | Tier 1 | Menstrual cycle phase reported but not controlled for within the study | 48 h |
| Avellini et al. [2] | F 4  M 4 | F 4 | 23.5 (±1.9) | 52.5 (±3.3) | Insufficient information to classify to a Tier | Pre and post acclimation testing completed pre and post ovulation | 5 days within the initiation of menstrual flow (pre-OV), or with the rise of early morning temperature (post-OV) |
| Barry et al. [3] ^a f^ | F 5  M 9 | F 5 | 27.0 (±5.0) | NS | Insufficient information to classify to a Tier | Menstrual cycle phase reported but not controlled for within the study | 12 - 48 h |
| Buono et al. [4] | F 20  M 13 | F 20 | 23.5 (±5.4) | NS | Insufficient information to classify to a Tier | No attempt to control for menstrual cycle phase | During last heat exposure |
| Campbell et al. [5] ^f^ | F 5  M 8 | F 5 | 23.0 (±4.0) | 54.0 (±8.0)  V̇O_2peak_ | Tier 1 | All testing and training occurred during 3-week active pill phase (all females using oral contraceptives) | During last heat exposure |
| Cleland et al. [6] | F 3 | F 3 | 26.7 (±9.9) | ~ 27.3 (±3.7) | Insufficient information to classify to a Tier | Menstrual cycle phase reported but not controlled for within the study | During last heat exposure |
| Cohen and Gisolfi [7] | F 12 | F 6 | 19.3 (±1.1) | 39.5 (±1.1) | Insufficient information to classify to a Tier | Menstrual cycle phase not controlled for | During last heat exposure |
| Fein et al. [8] | F 12 | F 12 | 21.8 (±2.8) | 40.2 (±5.5) | Insufficient information to classify to a Tier | Menstrual cycle phase reported but not controlled for within the study | During last heat exposure |
| Frye and Kamon [9] | F 4  M 4 | F 4 | 25.5 (±1.7) | 54.1 (±4.3) | Insufficient information to classify to a Tier | Pre and post acclimation testing aligned to phase of menstrual cycle | 24 h following last heat exposure (and/or until next phase of menstrual cycle) |
| Garrett et al. [10] | F 10 | F 10 | 22.6 (±2.7) | 43.9 (±8.6)  V̇O_2peak_ | Insufficient information to classify to a Tier | Heat tolerance testing (HST 2 and 3) completed in luteal phase of menstrual cycle or active pill phase for oral contraceptive users | 5 ± 2 days |
| Gore et al. [11] ^d e^ | F 17  M 45 | - | 21.1 (±4.5) | ~ 52.4 | Tier 3 | Menstrual cycle phase not controlled for | Within 7 days |
| Greenleaf et al. [12] | F 6  M 4 | F 6 | 22.0 (±1.0) | 47.0 (±3.0) V̇O_2peak_ | Insufficient information to classify to a Tier | Menstrual cycle phase not controlled for | During last heat exposure |
| Henderson et al. [13] ^d^ | F 19 | F 19 | 24.2 (± 4.4) | NS | Tier 4 | Menstrual cycle phase not controlled for | During last heat exposure |
| Horstman and Christensen [14] | F 4  M 6 | F 4 | 21.5 (±1.4) | 47.2 (±2.6) | Insufficient information to classify to a Tier | Menstrual cycle phase not controlled for | During last heat exposure |
| Kampmann et al. [15] ^e^ | F 2  M 6 | - | 27.9 (±3.4) | NS | Insufficient information to classify to a Tier | Menstrual cycle phase not controlled for | During last heat exposure |
| Kirby et al. [16] | F 8 | F 8 | 27.4 (±5.2) | 46.6 (±5.7)  V̇O_2peak_ | Tier 2 | Menstrual cycle phase reported but not controlled for within the study | During last heat exposure |
| Kirby et al. [17] ^a^ | F 16  M 10 | F 16 | 19.0 (±2.0) | 52.6 (±6.9) | Tier 2 | Identification, reporting and control of menstrual cycle within phase for most participants | After final heat exposure; ~ 28 days after baseline testing |
| Mee et al. [18] | F 8  M 8 | F 8 | 20.0 (±1.0) | ~ 45.7  V̇O_2peak_ | Insufficient information to classify to a Tier | Pre testing (RHTT 1 and 2) completed in follicular phase of menstrual cycle or day two of pill phase for oral contraceptive users. Post testing (RHTT 3) completed in luteal phase of self-reported menses. | 48 h |
| Mee et al. [19] ^a^ | F 9 | F 9 | 22.0 (±4.0) | 50.0 (±4.0) | Tier 2 | Pre and post acclimation testing completed in follicular phase of menstrual cycle | 24 h |
| Meylan et al. [20] ^d e^ | F 16 | - | 27.0 (±5.0) | 53.1 (±3.1) | Tier 4 | Menstrual cycle phase not controlled for | During last heat exposure |
| Moss et al. [21] | F 3  M 13 | F 3 | 32.7 (±7.6) | NS | Insufficient information to classify to a Tier | Menstrual cycle phase not controlled for | > 24 h |
| O’Toole et al. [22] | F 4 | F 4 | 26.5 (±4.4) | 48.0 (±7.6) | Insufficient information to classify to a Tier | Menstrual cycle phase reported but not controlled for within the study | During last heat exposure |
| Pethick et al. [23] ^e^ | F 18 | - | 27.3 (±3.2) | NS | Tier 4 | Menstrual cycle phase not controlled for | 4 days |
| Philp et al. [24] ^e f^ | F 3  M 8 | - | 21.0 (±3.0) | 61.4 (±5.1)  V̇O_2peak_ | Tier 3 | Menstrual cycle phase not controlled for | Within 5 days |
| Sawka et al. [25] (HA) ^e^ | F 9  M 31 | - | 22.0 (±3.0) | 40.0 (±5.0) | Insufficient information to classify to a Tier | Menstrual cycle phase not controlled for | 1 day |
| Sawka et al. [26] (Hypo) | F 6  M 6 | F 6 | 26.0 (±3.0) | 45.4 (±6.7) | Insufficient information to classify to a Tier | Menstrual cycle phase not controlled for | NS |
| Shapiro et al. [27] | F 9  M 10 | F 9 | 22.0 (±3.0) | 40.5 (±4.5) | Insufficient information to classify to a Tier | Menstrual cycle phase not controlled for | During last heat exposure |
| Stephenson et al. [28] ^a^ | F 7  M 22 | F 4 | 20.0 (±1.4) | 53.5 (±3.9)  V̇O_2peak_ | Tier 2 | Menstrual cycle phase not controlled for | TT completed 3 days post final heat exposure |
| Sunderland et al. [29] | F 17 | F 6 | 20.6 (±0.6) | 49.4 (±0.3) | Insufficient information to classify to a Tier | Performance testing completed in same phase of menstrual cycle | 48 h |
| Wyndham et al. [30] ^e^ | F 26  M 30 | - | NS | NS | Insufficient information to classify to a Tier | Menstrual cycle phase not controlled for | NS |

Data are presented as mean ± SD

*Key*: ^a^, includes passive heat exposure; ^b^, consecutive; ^c^, non-consecutive; ^d^, acclimatisation; ^e^, included in the systematic review only; ^f^ combined male and female data; *F* female, *h* hour, *M* male, *NS* not specified, *Pre-OV* pre-ovulation, *Post-OV* post-ovulation, *RHTT* running heat tolerance test, *TT* time trial, *V̇O_2max_* maximal oxygen uptake, *V̇O_2peak_* peak oxygen uptake

Athlete calibre has been retrospectively classified according to a recent framework [31]

**Reference List:**

1. Alkemade, P., et al. Individual characteristics associated with the magnitude of heat acclimation adaptations. Eur J Appl Physiol. 2021;1216:1593-1606; https://10.1007/s00421-021-04626-3.

2. Avellini, B.A., E. Kamon, and J.T. Krajewski. Physiological responses of physically fit men and women to acclimation to humid heat. J Appl Physiol Respir Environ Exerc Physiol. 1980;492:254-61; https://10.1152/jappl.1980.49.2.254.

3. Barry, H., et al. Improved neural control of body temperature following heat acclimation in humans. J Physiol. 2020;5986:1223-1234; https://10.1113/JP279266.

4. Buono, M.J., S. Leichliter Martha, and J.H. Heaney. Peripheral sweat gland function, but not whole-body sweat rate, increases in women following humid heat acclimation. J Therm Biol. 2010;353:134-137; https://10.1016/j.jtherbio.2010.01.004.

5. Campbell, H.A., et al. Acute physiological and psychophysical responses to different modes of heat stress. Exp Physiol. 2022;1075:429-440; https://10.1113/ep089992.

6. Cleland, T.S., S.M. Horvath, and M. Phillips. Acclimatization of women to heat after training. Int Z Angew Physiol. 1969;271:15-24; https://10.1007/BF00695014.

7. Cohen, J.S. and C.V. Gisolfi. Effects of interval training on work-heat tolerance of young women. Med Sci Sports Exerc. 1982;141:46-52; https://10.1249/00005768-198201000-00009.

8. Fein, J.T., E.M. Haymes, and E.R. Buskirk. Effects of daily and intermittent exposures on heat acclimation of women. Int J Biometeorol. 1975;191:41-52; https://10.1007/BF01459840.

9. Frye, A.J. and E. Kamon. Responses to dry heat of men and women with similar aerobic capacities. J Appl Physiol Respir Environ Exerc Physiol. 1981;501:65-70; https://https://doi.org/10.1152/jappl.1981.50.1.65.

10. Garrett, A.T., et al. Effectiveness of short-term heat acclimation on intermittent sprint performance with moderately trained females controlling for menstrual cycle phase. Front Physiol. 2019;10:1458; https://10.3389/fphys.2019.01458.

11. Gore, C.J., et al. VO2max and haemoglobin mass of trained athletes during high intensity training. Int J Sports Med. 1997;186:477-482; https://DOI: 10.1055/s-2007-972667.

12. Greenleaf, J.E., P.J. Brock, and D. Sciaraffa. Effects of exercise-heat acclimation on fluid, electrolyte, and endocrine responses during tilt and +Gz acceleration in women and men. Aviat Space Environ Med. 1985;567:683-689.

13. Henderson, M.J., et al. Responses to a 5-day sport-specific heat acclimatization camp in elite female rugby sevens athletes. Int J Sports Physiol Perform. 2022:1-10; https://10.1123/ijspp.2021-0406.

14. Horstman, D.H. and E. Christensen. Acclimatization to dry heat: active men vs. active women. J Appl Physiol Respir Environ Exerc Physiol. 1982;524:825-31; https://10.1152/jappl.1982.52.4.825.

15. Kampmann, B., et al. Lowering of resting core temperature during acclimation is influenced by exercise stimulus. Euro J Appl Physiol. 2008;1042:321-327; https://10.1007/s00421-007-0658-6.

16. Kirby, N.V., S.J.E. Lucas, and R.A.I. Lucas. Nine-, but not four-days heat acclimation improves self-paced endurance performance in females. Front Physiol. 2019;10MAY:539; https://10.3389/fphys.2019.00539.

17. Kirby, N.V., et al. Sex differences in adaptation to intermittent post-exercise sauna bathing in trained middle-distance runners. Sports Med Open. 2021;71:51; https://10.1186/s40798-021-00342-6.

18. Mee, J.A., et al. A comparison of males and females' temporal patterning to short- and long-term heat acclimation. Scand J Med Sci Sports. 2015;25 Suppl 1:250-8; https://10.1111/sms.12417.

19. Mee, J.A., et al. Sauna exposure immediately prior to short-term heat acclimation accelerates phenotypic adaptation in females. J Sci Med Sport. 2018;212:190-195; https://10.1016/j.jsams.2017.06.024.

20. Meylan, C.M., et al. The efficacy of heat acclimatization pre-world cup in female soccer players. Front Sports Act Living. 2021;3:116; https://https://doi.org/10.3389/fspor.2021.614370.

21. Moss, J.N., et al. Short-term isothermic heat acclimation elicits beneficial adaptations but medium-term elicits a more complete adaptation. Euro J Appl Physiol. 2020;1201:243-254; https://10.1007/s00421-019-04269-5.

22. O'Toole, M.L., et al. The effects of heat acclimation on plasma volume and plasma protein of females. Int J Sports Med. 1983;41:40-4; https://10.1055/s-2008-1026014.

23. Pethick, W.A., et al. The effect of a team sport-specific heat acclimation protocol on plasma volume in elite female soccer players. Sci Med Footb. 2018;21:16-22; https://10.1080/24733938.2017.1384559.

24. Philp, C.P., et al. Can ten days of heat acclimation training improve temperate-condition rowing performance in national-level rowers? PloS one. 2022;179:e0273909; https://10.1371/journal.pone.0273909.

25. Sawka, M.N., et al. Does heat acclimation lower the rate of metabolism elicited by muscular exercise? Aviat Space Environ Med. 1983;541:27-31.

26. Sawka, M.N., et al. Hypohydration and exercise: effects of heat acclimation, gender, and environment. J Appl Physiol Respir Environ Exerc Physiol. 1983;554:1147-53; https://10.1152/jappl.1983.55.4.1147.

27. Shapiro, Y., K.B. Pandolf, and R.F. Goldman. Sex differences in acclimation to a hot-dry environment. Ergonomics. 1980;237:635-42; https://10.1080/00140138008924778.

28. Stephenson, B.T., K. Tolfrey, and V.L. Goosey-Tolfrey. Mixed active and passive, heart rate-controlled heat acclimation is effective for paralympic and able-bodied triathletes. Front Physiol. 2019;10:1214; https://https://doi.org/10.3389/fphys.2019.01214.

29. Sunderland, C., J.G. Morris, and M.E. Nevill. A heat acclimation protocol for team sports. Br J Sports Med. 2008;425:327-33; https://10.1136/bjsm.2007.034207.

30. Wyndham, C.H., J.F. Morrison, and C.G. Williams. Heat reactions of male and female Caucasians. J Appl Physiol. 1965;203:357-64; https://10.1152/jappl.1965.20.3.357.

31. McKay, A.K.A., et al. Defining training and performance caliber: a participant classification framework*.* Int J Sports Physiol Perform. 2022;172:317-331; https://10.1123/ijspp.2021-0451.
