# Supplementary material for: Heat Adaptation for Females: A Systematic Review and Meta-Analysis of Physiological Adaptations and Exercise Performance in the Heat
Source: Sports Med. 2023 May 24;53(7):1395–421. doi: 10.1007/s40279-023-01831-2 (PMC10289939; doi:10.1007/s40279-023-01831-2)
Supplement: Supplementary file 7 — Supplementary file7 (DOCX 270 KB) [file 40279_2023_1831_MOESM7_ESM.docx]

**Online Resource 7**

**Title:** Heat Adaptation for Females: A Systematic Review and Meta-Analysis of Physiological Adaptations and Exercise Performance in the Heat.

**Journal**: Sports Medicine.

**Authors:** Monica K. Kelly^1^*, Steven J. Bowe^2,3^, William T. Jardine^1^, Dominique Condo^1^, Joshua H. Guy^4^, Rodney J. Snow^5^, and Amelia J. Carr^1^

^1^ Centre for Sport Research, Deakin University, 221 Burwood Highway, Burwood, VIC, 3125, Australia

^2^ Deakin Biostatistics Unit, Faculty of Health, Deakin University, 221 Burwood Highway, Burwood, VIC, 3125, Australia

^3^ Faculty and School of Health, Victoria University of Wellington, Kelburn Parade, Kelburn, Wellington, 6140, New Zealand

^4^ School of Health, Medical and Applied Sciences, Central Queensland University, Cairns, QLD, Australia

^5^ Institute for Physical Activity and Nutrition, Deakin University, 221 Burwood Highway, Burwood, VIC, 3125, Australia

**Corresponding author**: Monica Kelly ([monica.kelly@research.deakin.edu.au](mailto:monica.kelly@research.deakin.edu.au))

**Electronic Supplementary Material Appendix S7.**

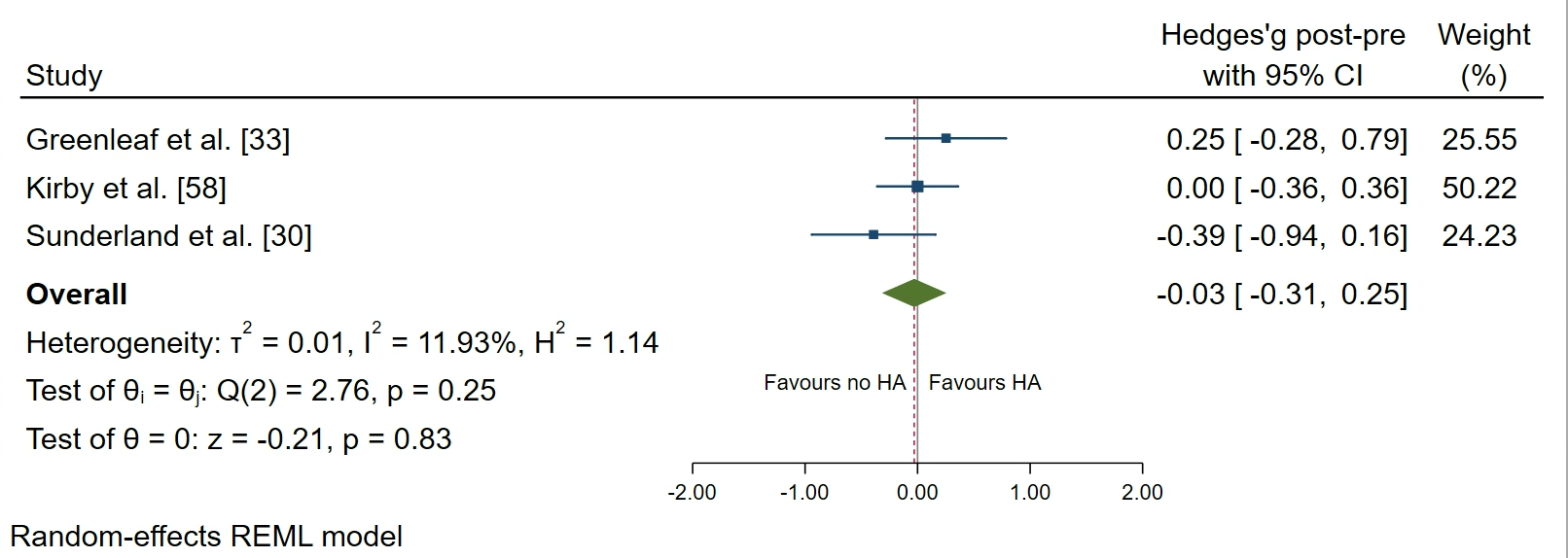


**Fig. S1** Plasma volume forest plot. Data are presented as Hedges’ g and 95% confidence intervals. Effects to the left of 0 (solid line) indicate a reduction in plasma volume, whereas effects to the right of 0 indicate an increase in plasma volume with heat adaptation regimens. The red dotted line indicates the effect line of included studies within the Forest plot
